# Supplementary material for: Simultaneously determining regional heterogeneity and connection directionality from neural activity and symmetric connection
Source: PLoS Comput Biol. 2025 Oct 23;21(10):e1013612. doi: 10.1371/journal.pcbi.1013612 (PMC12574905; doi:10.1371/journal.pcbi.1013612)
Supplement: S1 Text — S1.1 Resimulation of Model A. S1.2 Mapping of Heterogeneity Pairs – Model A and Model B. S1.3 Implement of Feedback Inhibition Control. S1.4 Resimulation of Model C. (DOCX) [file pcbi.1013612.s001.docx]

**Simultaneously Determining Regional Heterogeneity and Connection Directionality from Neural Activity and Symmetric Connection**

**Jiawen Chang^1,2^, Zhuda Yang^1,2^, Changsong Zhou^1,2,3*^**

**S1** **Text. Supplementary Methods**

**S1.1 Resimulation of Model A**

**S1.2 Mapping of Heterogeneity Pairs – Model A and Model B**

**S1.3 Implement of Feedback Inhibition Control**

**S1.4 Resimulation of Model C**

**S1 Text. Supplementary Methods**

**S1.1 Resimulation of Model A with reconstructed parameters**

To quantify how well the estimated effective heterogeneity $\hat{h}_{i}$ and asymmetric SC $\hat{C}_{ij}$ can reproduce the original FC patterns, we simulated linear dynamics (Eq. 4) using the estimated effective heterogeneity $\hat{h}_{i}$ and asymmetric SC $\hat{C}_{ij}$, and compared its resulting FC patterns with those from the original neural activity (Figure S4B, Dashed line). Results show that with increasing G, the effective heterogeneity and asymmetric SC successfully reproduced the FC patterns, consistent with the decreasing trend of REs observed in the main text (Figure 2A-C). These findings demonstrate that our framework can effectively separate neural activity into effective heterogeneity and asymmetric SC components while preserving the essential FC patterns.

The linear model is similar to Eq.4 but $J(S^{*})$ is replaced by our estimated features:

$$\begin{aligned} \frac{dS}{dt}=J_{resim}S+\sigma\xi,\#\left( S1 \right) \end{aligned}$$

where

$$J_{resim}=\left\{ \begin{aligned} \left[ \hat{J} \right]_{ii}=\left[ <\frac{dS}{dt},S>{<S,S>}^{-1} \right]_{ii},i=j \\ \hat{h}_{i}\hat{C}_{ij},i\neq j \end{aligned}. \right.$$

To quantify how well the heterogeneous parameters $\hat{w}_{i}$ and $\hat{I}_{i}$ reconstructed from effective heterogeneity $\hat{h}_{i}$ can reproduce the original FC patterns, we simulated Model A (Eqs.1-3) using these estimated parameters and estimated asymmetric SC, and compared its resulting FC patterns with those from the original neural activity (Figure S4B, Solid line). Notably, despite the challenges in estimating heterogeneous parameters near the second bifurcation point (G=1.3) where the system exhibits highly nonlinear behavior (Figure 3B), our framework - though based on linear assumptions in the asynchronous state - still achieves a reasonable average correlation of 0.84. This robust performance in the highly nonlinear regime suggests that our method can effectively capture essential FC characteristics even when operating beyond its underlying linear assumptions.

**S1.2 Mapping of Heterogeneity Pairs – Model A and Model B**

To demonstrate that our effective heterogeneity $h_{i}$ serves as a unified framework for quantifying how different heterogeneous parameters contribute to EC directionality, we analytically derived the mapping relationship between two distinct parameter pairs: $w-I$ heterogeneity in Model A and $\tau-b$ heterogeneity in Model B. This mapping not only reveals how different parameter combinations can generate similar dynamics, but also provides ground truth calculations for $\tau_{i}$ and $b_{i}$, further validating the generality of our framework in capturing heterogeneity-induced directional effects.

In $J_{B}$ of Eq. 34, $H_{i}$ is heterogeneous across ROIs because of $b_{i}$ and we defined $x_{i}^{'}=wJ_{N}S_{i}^{*}+GJ_{N}\Sigma_{j}C_{ij}S_{j}^{*}+I$. We assume the Jacobian matrix in Eq. 27 is equal to Jacobian matrix of Eq. 34 to make sure both models reach the same level of stability and have a similar FC pattern around the stable state according to Lyapunov equation. Therefore, we can obtain the following equations:

$$\left\{ \begin{aligned} -\frac{1}{\tau_{i}\left( 1-S_{i}^{*} \right)}+wrJ_{N}\left( 1-S_{i}^{*} \right)\frac{\partial H}{\partial x_{i}}\left. \right|_{x_{i}=x_{i}^{*}}=\frac{1}{\tau_{S}\left( 1-S_{i}^{*} \right)}+w_{i}rJ_{N}\left( 1-S_{i}^{*} \right)\frac{\partial H}{\partial x_{i}}\left. \right|_{x_{i}=x_{i}^{*}}, \\ \frac{\partial H_{i}}{\partial x_{i}}\left. \right|_{x_{i}=x_{i}^{'}}=\frac{\partial H}{\partial x_{i}}\left. \right|_{x_{i}=x_{i}^{*}}. \end{aligned} \right.$$

By solving these equations, we obtain the relationship of the $w-I$ heterogeneity and $\tau-b$ heterogeneity:

$$\begin{aligned} \left\{ \begin{aligned} \frac{1}{\tau_{i}}=\frac{1}{\tau_{S}}-\left( w_{i}-w \right)rJ\left( 1-S_{i}^{*} \right)^{2}\frac{\partial H}{\partial x_{i}}\left. \right|_{x_{i}=x_{i}^{*}}, \\ b_{i}=b-aJ\left( w_{i}-w \right)S_{i}^{*}-a\left( I_{i}-I \right). \end{aligned} \right.\#\left( S2 \right) \end{aligned}$$

We use *fsolve.m* in MATLAB to solve $b_{i}$ from the derivative of activation function:

$$\begin{aligned} \frac{\partial H_{i}}{\partial x_{i}}\left. \right|_{x_{i}=x_{i}^{'}}-\frac{\partial H}{\partial x_{i}}\left. \right|_{x_{i}=x_{i}^{*}}=0.\#\left( S3 \right) \end{aligned}$$

**S1.3 Implement of Feedback Inhibition Control**

Feedback inhibition control (FIC) was introduced as a local regulatory mechanism to maintain neural population stability in large-scale brain networks [1-3]. This approach compensates for excessive excitatory activity by dynamically adjusting the inhibitory-to-excitatory connection weights within each cortical region, effectively clamping the firing rate of local excitatory populations at a target level (typically 3 Hz). By implementing this homeostatic control mechanism, FIC ensures that neural populations operate within physiologically realistic firing rate ranges while preserving the network's capacity for information transmission and processing.

Here, we use two E-I models to test our reconstruction framework (Eqs.37-42) with (change regional $w_{EI}^{i}$ ensuring regional firing rate $H\left( x_{E}^{i} \right)=3Hz$) or without FIC ($w_{EI}=2$ identical across each region).

The FIC is implemented by solving Eqs. 37-42 at every parameter set $\{G,w_{EE}^{i},w_{IE}^{i}\}$. Start from Eq.37 and 39, the requirement of regional firing rate $H\left( x_{E}^{i} \right)\approx3Hz$ at steady state ($\frac{dS_{E}}{dt}=0$) implies that $S_{E}^{i*}\approx0.164757$and $x_{E}^{i*}\approx0.37738 nA$ [3]. We next solve the overall inhibition external input $x_{I}^{i*}$ by first reforming Eq.42:

$$S_{I}^{i*}=w_{IE}^{i}S_{E}^{i*}-x_{I}^{i*}+I_{I},$$

then letting Eq.38 equals to zero and we have:

$$-\frac{w_{IE}^{i}S_{E}^{i*}-x_{I}^{i*}+I_{I}}{\tau_{I}}+H_{I}\left( x_{I}^{i*} \right)=0,$$

where we can solve $x_{I}^{i*}$ because all other values are given, then solve $S_{I}^{i*}$ according to Eq.41.

Finally, we can solve regional $w_{EI}^{i}$:

$$\begin{aligned} w_{EI}^{i}=\frac{w_{EE}^{i}S_{E}^{i*}+G\Sigma_{j}C_{ij}S_{E}^{j*}-x_{E}^{i*}+I_{E}}{S_{I}^{i*}}.\#\left( S4 \right) \end{aligned}$$

We note that, during the FIC procedure, only global coupling level $G$, heterogeneous E-to-E strength $w_{EE}^{i}$ and E-to-I strength $w_{IE}^{i}$ are needed [3].

**S1.4 Resimulation of Model C with reconstructed parameters**

To validate whether the effective heterogeneity and asymmetric SC identified by our reconstruction framework using only the excitatory population from E-I models can sufficiently capture FC patterns, we simulated a linear model (Eq.10) and compared its FC with that of the original E-I model's excitatory population (Eq.37-42). This comparison allows us to assess whether the reconstructed parameters contain the essential information needed to reproduce the observed functional dynamics, thereby confirming the adequacy of our simplified reconstruction approach in capturing the underlying network properties that drive FC patterns.

The linear model is similar to Eq.10 but $J_{eff}$ is replaced by our estimated features:

$$\begin{aligned} \frac{dS}{dt}=J_{resim}S+\sigma\xi,\#\left( S5 \right) \end{aligned}$$

where

$$J_{resim}=\left\{ \begin{aligned} \left[ \hat{J}_{eff} \right]_{ii}=\left[ <\frac{dS_{E}}{dt},S_{E}>{<S_{E},S_{E}>}^{-1} \right]_{ii},i=j \\ \hat{h}_{i}\hat{C}_{ij},i\neq j \end{aligned}. \right.$$

**Reference**

[1] Deco G, Ponce-Alvarez A, Hagmann P, Romani GL, Mantini D, Corbetta M. How local excitation–inhibition ratio impacts the whole brain dynamics. Journal of Neuroscience. 2014 Jun 4;34(23):7886-98.

[2] Deco G, Kringelbach ML, Arnatkeviciute A, et al. Dynamical consequences of regional heterogeneity in the brain's transcriptional landscape. Sci Adv. 2021;7(29):eabf4752.

[3] Demirtaş M, Burt JB, Helmer M, et al. Hierarchical heterogeneity across human cortex shapes large-scale neural dynamics. Neuron. 2019;101(6):1181-1194.

[4] Gilson M, Moreno-Bote R, Ponce-Alvarez A, Ritter P, Deco G. Estimation of directed effective connectivity from fMRI functional connectivity hints at asymmetries of cortical connectome. PLoS computational biology. 2016 Mar 16;12(3):e1004762.
