# Supplementary figures and images for: Simultaneously determining regional heterogeneity and connection directionality from neural activity and symmetric connection
Source: PLoS Comput Biol. 2025 Oct 23;21(10):e1013612. doi: 10.1371/journal.pcbi.1013612 (PMC12574905; doi:10.1371/journal.pcbi.1013612)

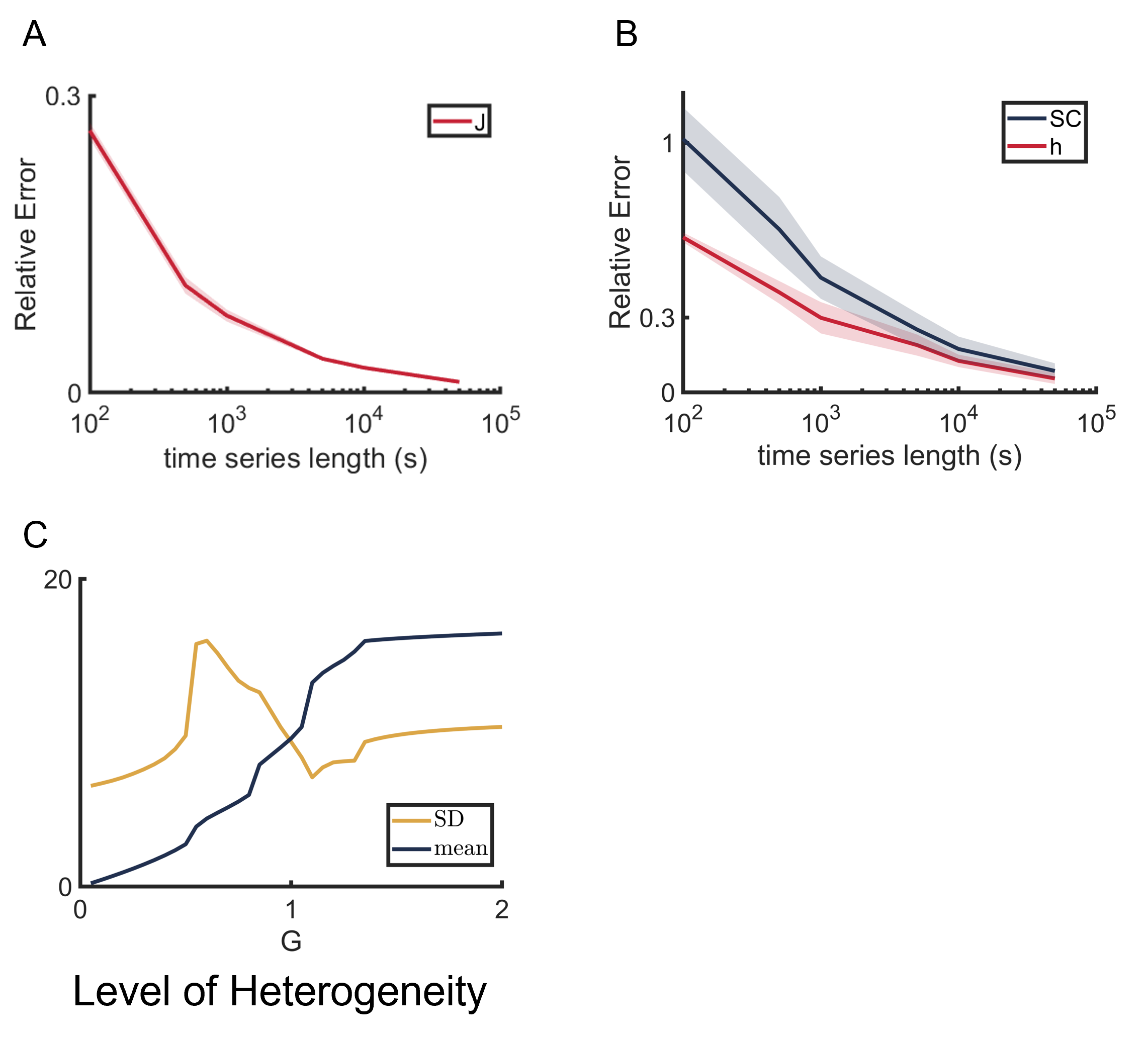

Supplement: S1 Fig — (A) The relative errors between ground truth and estimation of Jacobian as functions of data length. (B) The relative errors of estimated asymmetric SC (blue) and regional heterogeneity hi (red) as functions of data length. (C) The mean and SD of effective heterogeneity hi as functions of G. The mean of hi monotonically increases as G increases, while SD of hi shows a non-monotonous shape (first increase then decrease). (TIF) [file pcbi.1013612.s002.tif]

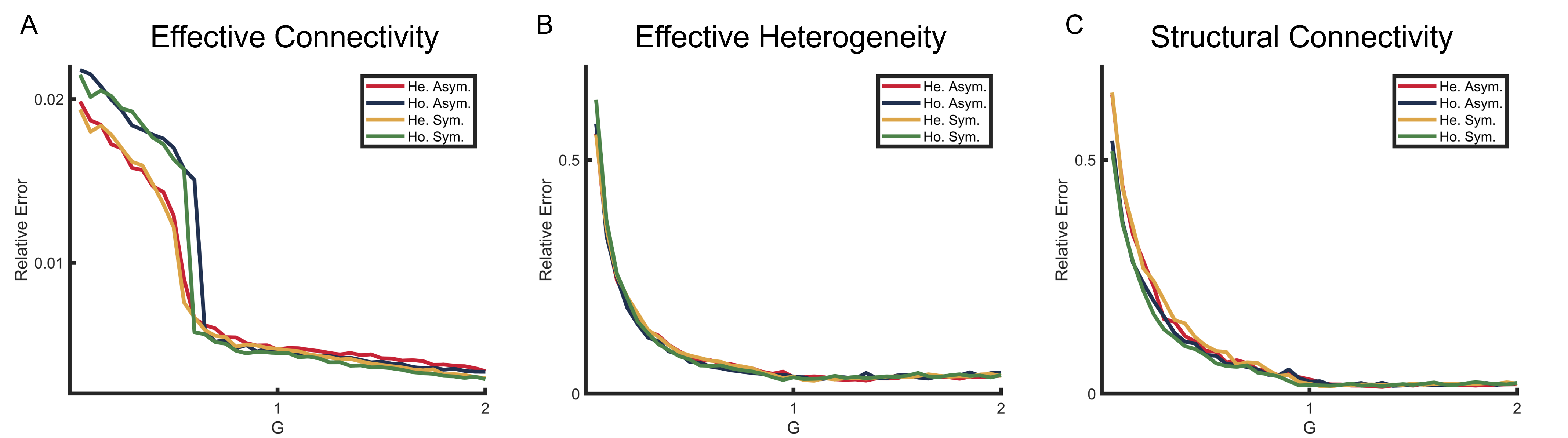

Supplement: S2 Fig — The relative errors of EC (A), effective heterogeneity (B) and asymmetric SC (C) reconstruction across four conditions where there is no ground truth asymmetry or heterogeneous parameter. All four conditions show comparable performance across different ground truth conditions as the global coupling G increases. Four ground truth models are considered for simulations: Heterogeneous parameters and asymmetric SC (He. Asym.); Homogeneous parameters and asymmetric SC (Ho. Asym.); Heterogeneous parameters and symmetric SC (He. Sym.); Homogeneous parameters and symmetric SC (Ho. Sym.). Heterogeneous parameters are wi and Ii of Model A in the main text, homogeneous parameters are defined as the mean of wi and Ii across regions. (TIF) [file pcbi.1013612.s003.tif]

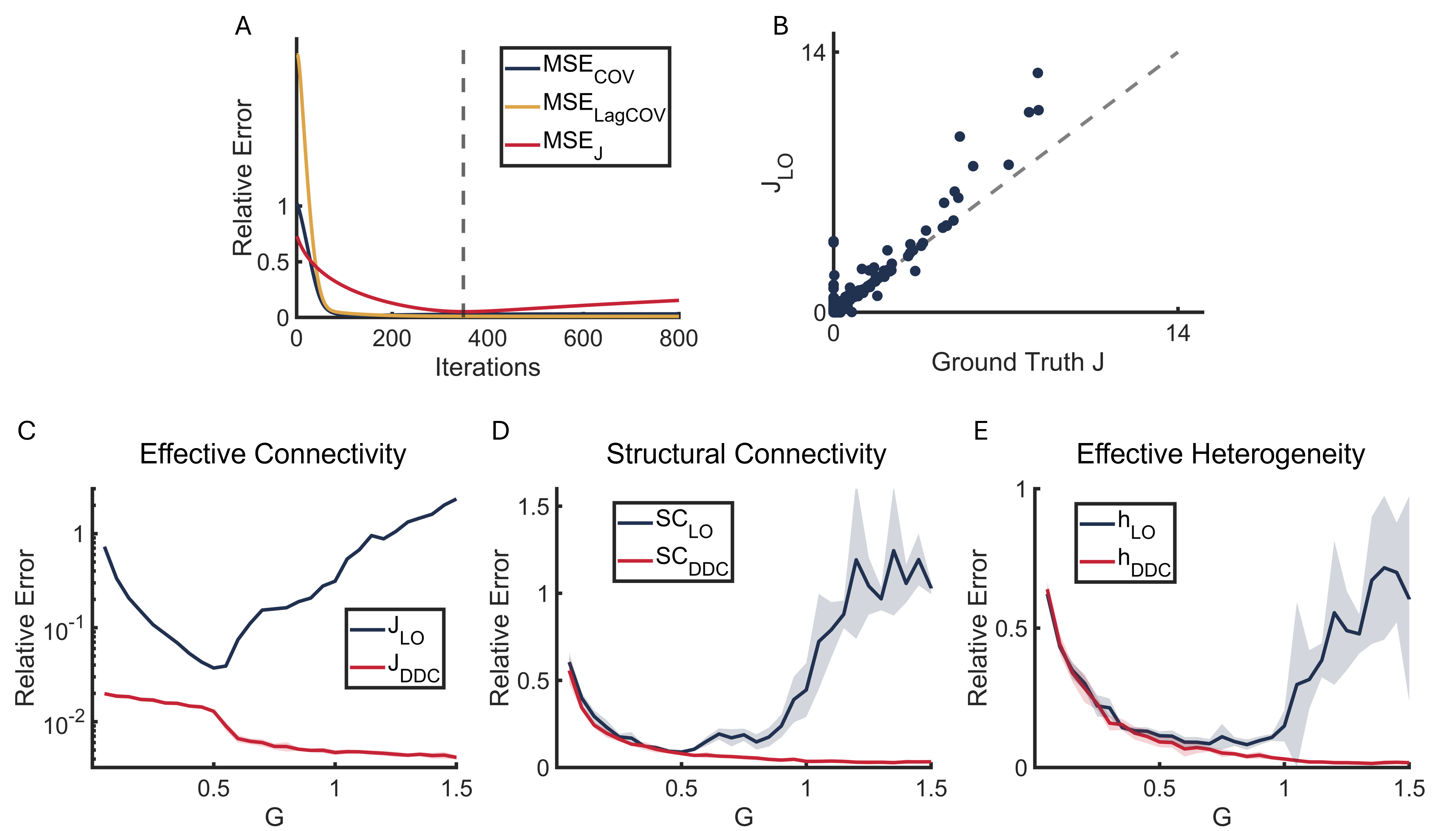

Supplement: S3 Fig — (A) Example of iterative minimization of zero-lag and tau-lag covariance of neural activity through Lyapunov Optimization (LO) [4]. Color lines are the mean square error (MSE, defined as the Frobenius norm between estimate and ground truth as original research [4]) of the zero-lag covariance (blue), tau-lag covariance (yellow) and EC (red). (B) Reconstruction performance of estimating EC, where JLO represents estimation using LO method. (C-E) Comparison of DDC and LO methods for estimating EC (C), effective heterogeneity (D), and asymmetric SC (E) across different G values. Our framework of separating EC into effective heterogeneity and asymmetric SC components demonstrates robust performance even under challenging conditions (e.g., G = 1) where LO encounters convergence difficulties. Tau-lag is chosen equal to τs. Shaded color represents 1-SD range across 10 times repetition. (TIF) [file pcbi.1013612.s004.tif]

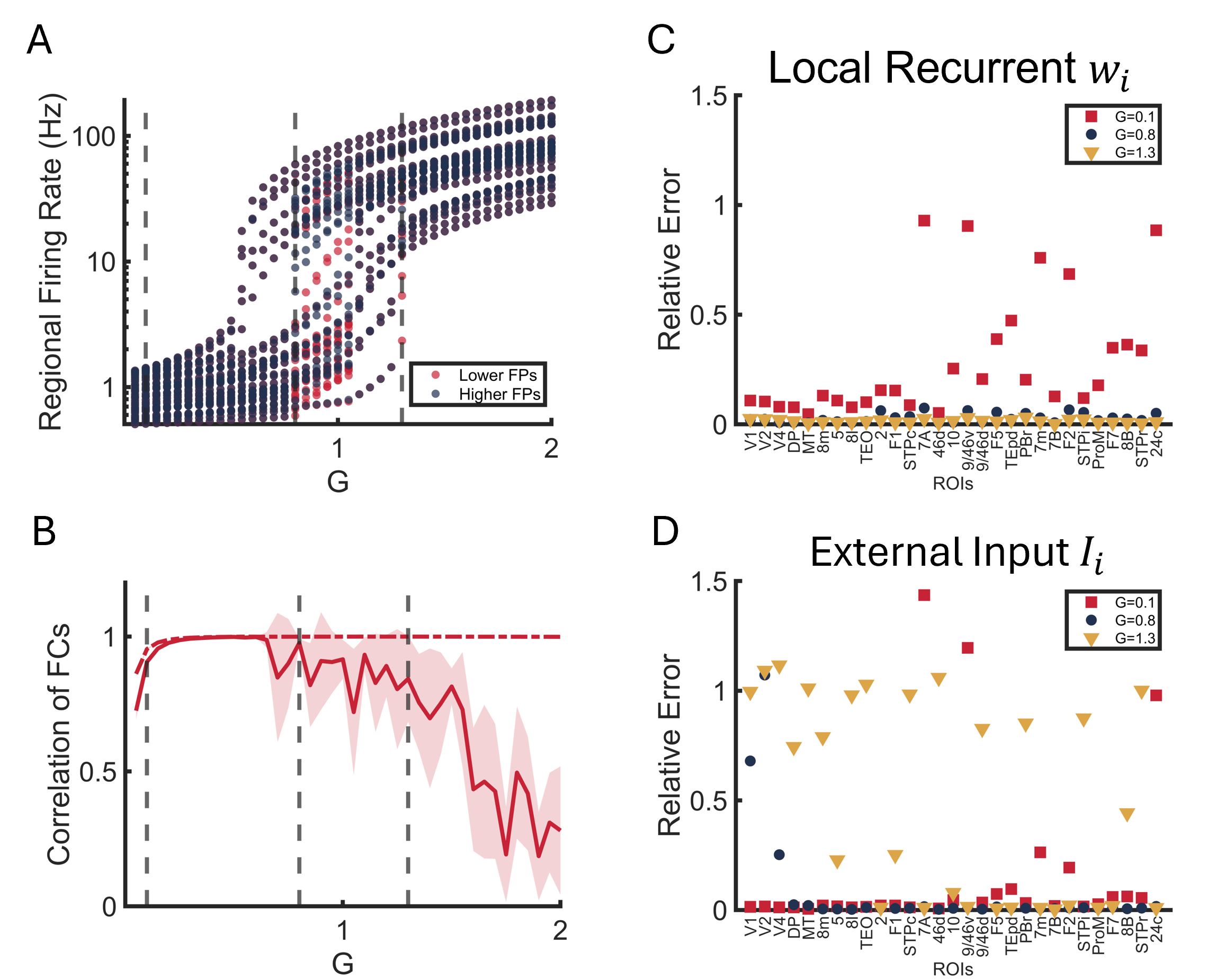

Supplement: S4 Fig — (A) Bistability across G. Each dot represents the stable firing rate of single region at every G. Red (Blue) dots are fixed points (FP) that start from lower (higher) initial states, representing lower (higher) stability. (B) Correlation between re-simulated FC and original FC. Solid line represents re-simulation of the model using reconstructed wi and Ii and reconstructed asymmetric SC. Dash line represents re-simulation using effective heterogeneity and reconstructed asymmetric SC (Eq. S1). (C and D) Relative error of wi and Ii across regions at three global coupling strength. (TIF) [file pcbi.1013612.s005.tif]

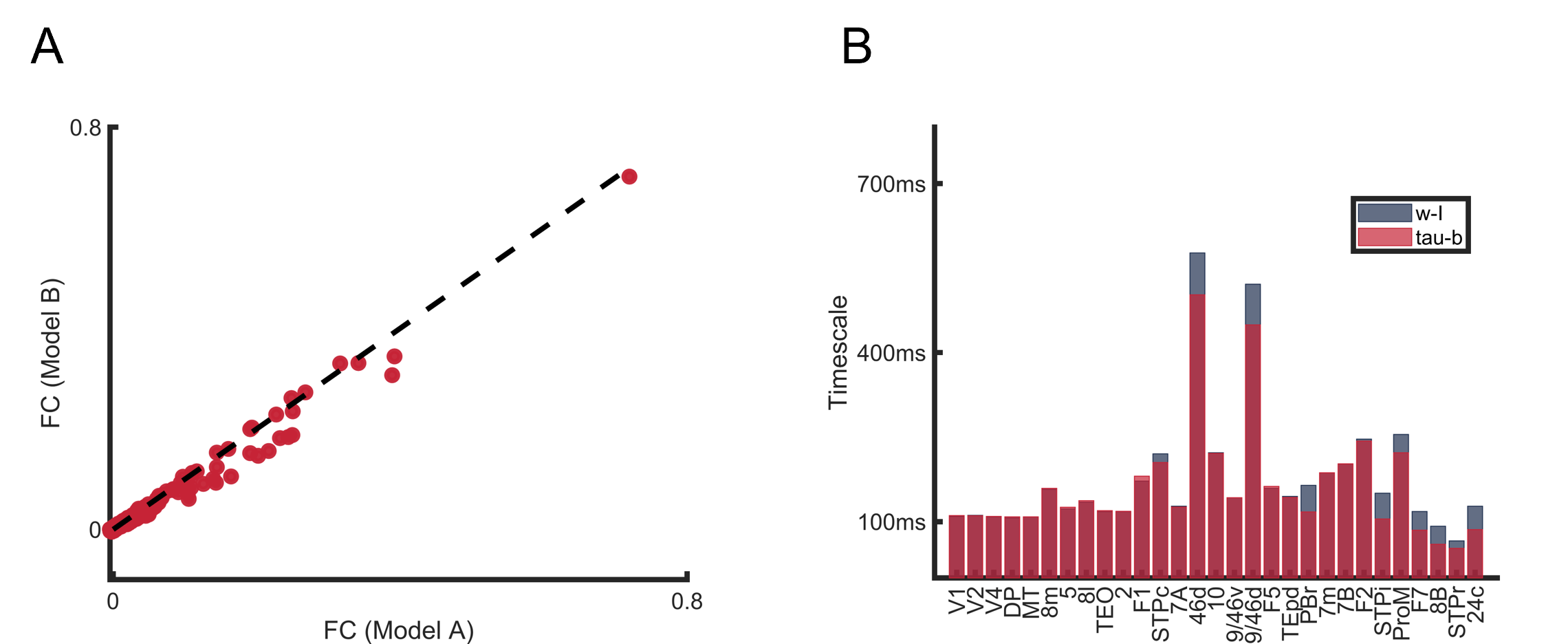

Supplement: S5 Fig — (A) Element-wise comparison between simulated FC from model A (heterogeneous wi and Ii) and FC from model B (heterogeneous τi and bi). (B) The resting state autocorrelation timescales of model A (blue) and model B (red) across ROIs. Here, we choose G = 0.7 as an example. (TIF) [file pcbi.1013612.s006.tif]

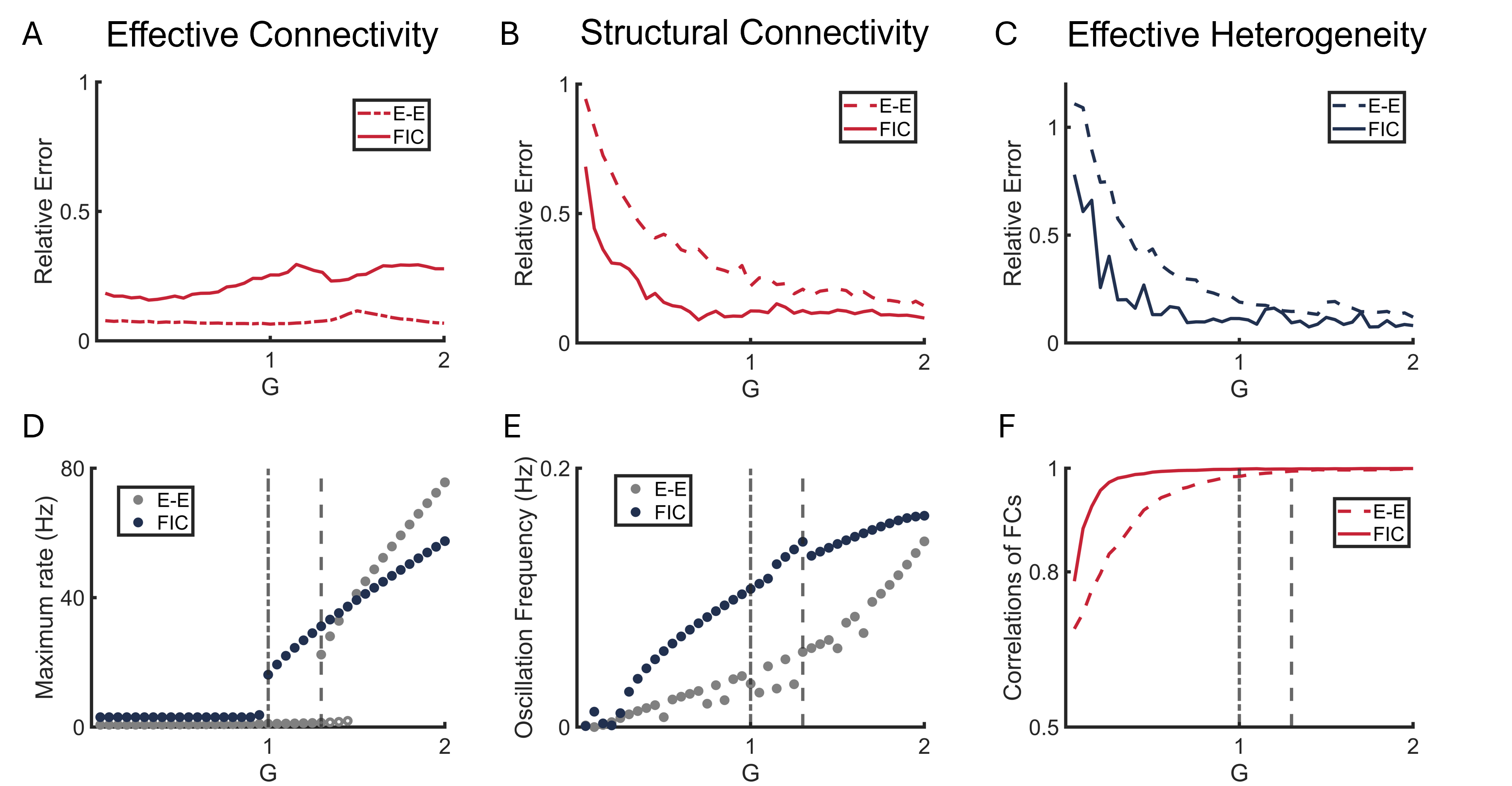

Supplement: S6 Fig — (A-C) Reconstruction of EC (A), effective heterogeneity (B) and asymmetric SC (C) using excitatory population activity simulated from E-I model with (solid line) or without FIC (dashed line). (D) Bifurcations of E-I models. Dashed line indicates where the E-I model exhibits a second fixed point (hollow gray circles). Dot-dashed line represents FIC instability, where firing rates cannot be controlled at 3Hz. (E) Intrinsic oscillation frequency calculated from imaginary eigenvalues divided by 2π. Eigenvalues are calculated by the Jacobian matrix at the fixed points. Dashed and dot-dashed lines correspond to the same conditions as in (D). (F) Correlations of FC between signals resimulated using estimated effective heterogeneity and asymmetry (Eq. S5) versus FC calculated from E population activity of the ground truth model. (TIF) [file pcbi.1013612.s007.tif]

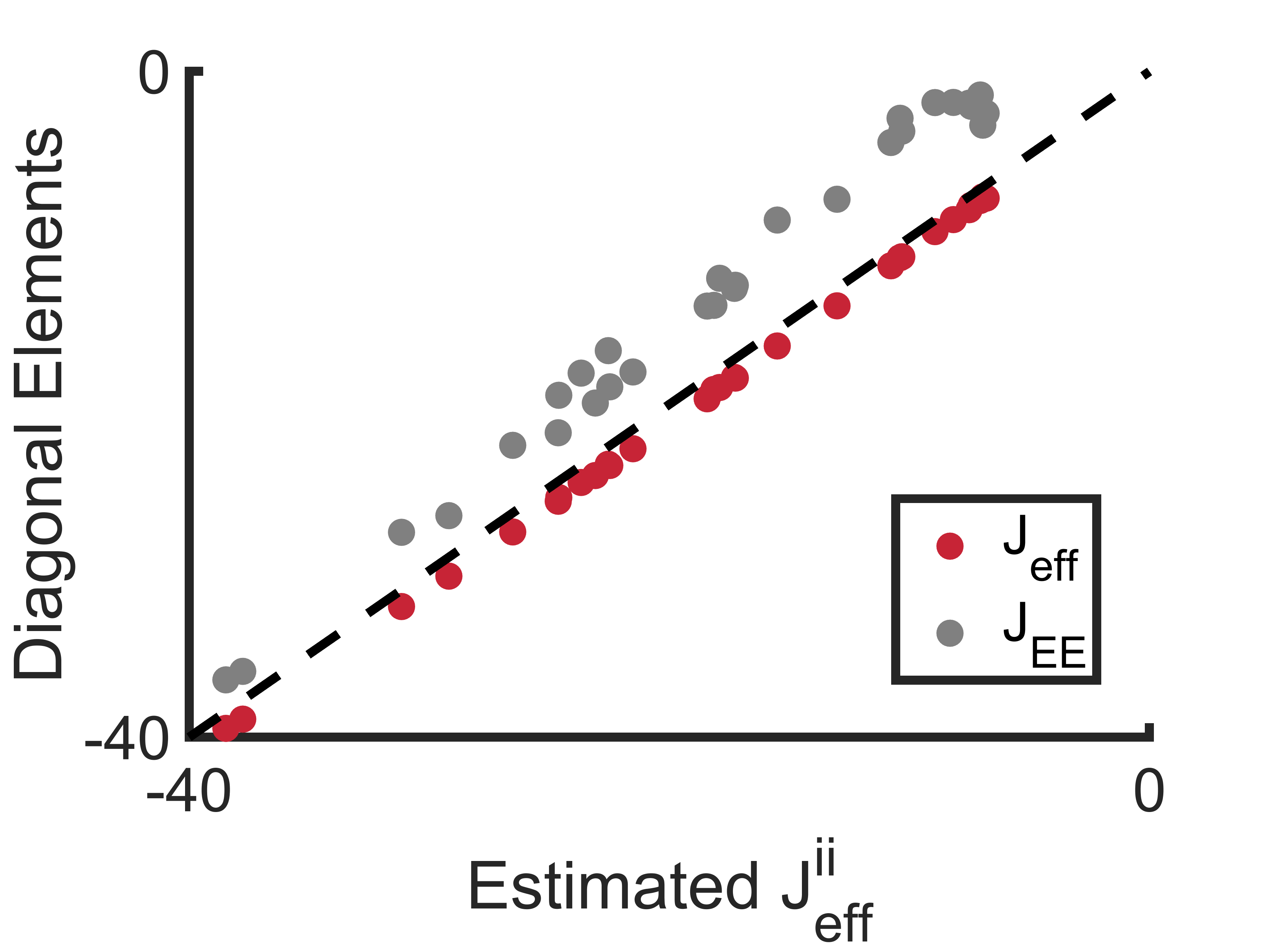

Supplement: S7 Fig — Diagonal elements comparison for JEE and modified effective Jacobian Jeff with estimated couplings J^E from the neural activity of excitatory population only. Here, we choose G = 1.1 as an example. (TIF) [file pcbi.1013612.s008.tif]
