# Supplementary material for: Simultaneously determining regional heterogeneity and connection directionality from neural activity and symmetric connection
Source: PLoS Comput Biol. 2025 Oct 23;21(10):e1013612. doi: 10.1371/journal.pcbi.1013612 (PMC12574905; doi:10.1371/journal.pcbi.1013612)
Supplement: Attachment — Submitted filename: PCOMPBIOL-D-25-00885 review.docx [file pcbi.1013612.s009.docx]

1. Summary

This manuscript introduces a novel framework to simultaneously reconstruct regional heterogeneity and asymmetric connections in brain networks using neural activity and symmetric structural connectivity data — both commonly available in experimental settings like fMRI and diffusion MRI.

The method builds upon and extends the Dynamical Differential Covariance (DDC) approach, which was previously developed for homogeneous networks. The authors demonstrate that regional heterogeneity and directionality can be separated mathematically and inferred via temporal reconstruction (estimating the Jacobian from neural dynamics, decoding dynamical directionality) followed by spatial reconstruction (decomposing the Jacobian using symmetric connectivity constraints, decoding effective heterogeneity and asymmetric structural connectivity). They validate the method using realistic macaque brain network models, including excitatory-inhibitory population dynamics.

The reviewer thinks the findings are suitable for publication in the PLoS Computational Biology, and sophisticated calculations are mostly carried out thoroughly and well described. The reviewer has only a few points for the improvement of the manuscript.

2. Major Points

A) Introduction: The Introduction is thoroughly written, providing enough motivation for the methods. Would you just provide why you chose to based your development on DDC method? In other words, what was the advantage the original DDC has over other EC methods?

B) Results: The result section is rich with extensive models. I do have following suggestions.

i) For a broader neuroscience audience, would you elaborate a bit more on the meaning of Jacobian structure and its implications? Also, citing one or two references (perhaps textbooks on dynamical systems or nonlinear dynamics) in the calculation of Jacobian (eq. 5 and on) would be helpful for the novices in the dynamical systems approach to the neuroscience.

ii) I don’t have a clear idea, but I feel Figure 1 could be improved to include more insights on the temporal / spatial reconstruction scheme, especially the spatial reconstruction.

iii) Would you describe at least briefly, how you arrived at the specific parameters on the table 2, or perhaps provide a very brief summary of the biological meanings for the values of parameters?

iv) For the defined rho square in line 313 of page 19, would you at least provide a very brief summary of what the definition means for self-confinement of the manuscript, rather than just referring to reference 44?

v) I am probably missing something, would you provide a bit more detail on how you got to eq. 18 from eq. 16?

vi) In line 494 of page 29, I know reference 38 is referred to before, but would you cite it again as the source of the model C and eqs 23-28?

C) Discussion: I feel that the discussion part is thoughtfully written and has wide-ranging implications discussed well.

i) I just have one question. Would the findings and analysis schemes in this manuscript help in anyway to understand and analyze rapid BOLD dynamics, i.e., directionality changes between top-down pathway (information flow from higher order regions to lower order sensory area) and bottom-up pathway (Honey et al., 2017, Network Neuroscience 1, 339; or perhaps Bolt et al, 2022, Nature Neuroscience 25, 109)?
