# Supplementary material for: Simultaneously determining regional heterogeneity and connection directionality from neural activity and symmetric connection
Source: PLoS Comput Biol. 2025 Oct 23;21(10):e1013612. doi: 10.1371/journal.pcbi.1013612 (PMC12574905; doi:10.1371/journal.pcbi.1013612)
Supplement: Attachment — Submitted filename: Response to Reviewers.docx [file pcbi.1013612.s011.docx]

Dear Editors,

Thank you for your editorial advice on our manuscript COMPBIOL-D-25-00885
“Simultaneously Determining Regional Heterogeneity and Connection Directionality from Neural Activity and Symmetric Connection” to PLOS Computational Biology.

We thank the reviewers for their constructive comments and suggestions. We have considered all of them carefully and addressed them in the revised manuscript. Below please find our point-by-point response to the reviewers.

Thank you for your attention and we look forward to your further editorial advice.

Sincerely yours,

Changsong Zhou (on behalf of the co-authors)

Email: [cszhou@hkbu.edu.hk](mailto:cszhou@hkbu.edu.hk)

Chair Professor, Department of Physics,

Director, Center for Nonlinear Studies

Director, Life Science Imaging Centre

Deputy Director, Institute of Computational and Theoretical Studies

Hong Kong Baptist University

Google Scholar:

<https://scholar.google.com.hk/citations?user=p3V8N-4AAAAJ&hl=en>

**Response to Reviewers**

**General Response**

We are grateful to the reviewers for their constructive feedback and valuable insights. In response to their feedback, we have conducted supplementary analyses, clarified key methodological details, updated the results and strengthened the overall narrative of the manuscript.

Below we provide detailed point-by-point responses to each reviewer's comments. For questions that were raised by multiple reviewers or contained multiple components, we have structured our responses to address each concern comprehensively while avoiding redundancy.

The major improvements include:

- New experiments on testing the limitations of our reconstruction framework. These results are updated in the Main Text and Supplemental Information.
- Reorganization of sections and figures in the Main Text for clearer illustration.
- Thoroughly revision of manuscript to improve the clarity of writing.

The specific revisions addressing each reviewer's concerns are outlined as follows:

**Overall Response to Baseline/Benchmark Comparisons**

We have addressed the issue of relative error evaluation and baseline comparison through systematic analyses in three complementary approaches:

**First (Figure 2):** We systematically tested the consequences of ignoring key model features. Using neural activity simulated from models with heterogeneity and asymmetric connections as ground truth, we compared reconstruction performance under different assumptions: When the ground truth asymmetry is ignored, systematic errors reach ~0.5. When the ground truth effective heterogeneity is ignored, systematic errors are substantially larger than our full framework, while our framework typically achieves <0.2 at non localized states ($G>0.3$). These comparisons demonstrate that accounting for both features provides meaningful and quantifiable improvements.

**Second (Figure S2):** We tested whether our framework can correctly identify the directionality of effective connectivity contributed by heterogeneity and/or asymmetry. We simulated neural activity under four conditions: heterogeneous parameters and asymmetry (He. Asym.), heterogeneous parameters and symmetry (He. Sym,), homogeneous parameters and asymmetry (Ho. Asym.), and homogeneous parameters and symmetry (Ho. Sym.). All four conditions were then analyzed using our complete reconstruction framework (Eqs. 7-9). Results show comparable performance across all these ground truth conditions as global coupling strength increases, confirming our framework's ability to recover true effective heterogeneity and asymmetric connectivity. This analysis differs from the first: in the first analysis, we deliberately ignored features during reconstruction while the neural activity inherently contained both heterogeneity and asymmetry; here, the reconstruction framework always considers both features, but the ground truth neural activity may or may not contain heterogeneity and/or asymmetry.

**Third (Figure S3):** We explored an alternative approach alongside our DDC-based method: Lyapunov optimization (LO) that optimizes effective connectivity by minimizing neural activity covariance and time-lagged covariance errors [53]. Our framework demonstrates robustness that is relatively independent of the specific EC estimation method employed. Notably, our approach maintains good performance even under challenging conditions (e.g., G=1) where LO encounters convergence difficulties, suggesting that DDC provides computational advantages for our temporal reconstruction framework.

Collectively, these systematic comparisons clearly demonstrate the added value and performance of our heterogeneity-aware framework.

**Response to Reviewer #1:**

The manuscript by Chang and colleagues introduces a computation framework to estimate regional heterogeneity along with the directed connectivity from observed data. Specifically, they extend an existing framework that estimates the directed connections to include regional heterogeneity. Estimating the regional heterogeneity and directed (effective) connectivity is an important issue in large-scale computational modeling. Although in some studies the problem was highlighted, to my knowledge there are no studies directly attempting to address this issue. The overall methodological approach in the manuscript is sound. However, the authors need to resolve some major issues that can be categorized into 3 groups. First, the overall organization of the manuscript requires major revision, particularly to include all details to replicate the study. Second, although presented results show that the framework is robust, some critical questions to support the claims of the manuscript remains unanswered. Third, some of the claims in the discussion are not backed and limitations of the framework should be better emphasized in the manuscript.

**Response:** Thank you for your positive evaluation of our work and valuable suggestions for revision.

**Major Comments:**

**Q1:** Throughout the manuscript, relative error is used to quantify how well the framework performs. Although this quantity alone gives an idea about the relative performance of the fitting under different conditions, it lacks any proper reference. How the authors define the relative error good enough to support their claims? As far as I see, there are substantial differences in the scales of y-axis across different figures. The authors could use simplified models such as symmetrical and/or homogenous regional parameters as a baseline comparison. Optionally, it would be beneficial to compare the framework to more straight-forward ones such as heuristic approaches to estimate effective connectivity and heterogeneity in terms of recovery of ground truth parameters and computational cost.

**A1:** Thank you for these constructive suggestions. Following your suggestion, we have addressed the baseline comparison concerns raised here in our "**Overall Response to Baseline/Benchmark Comparisons**" section above, which provides systematic comparisons using three complementary approaches that establish clear baselines and demonstrate the added value of our framework. Regarding the substantial differences in y-axis scales across figures, these reflect the inherent differences in parameter ranges and sensitivity across different network properties (e.g., heterogeneity level, connectivity constraints and noise strength), and our baseline comparisons now provide the necessary reference points to interpret these relative error magnitudes meaningfully. The method details are presented in [Materials and Method].

Comparing different heuristic approaches such as Partial Correlation, Granger Causality and Transfer Entropy is an excellent idea to illustrate the performance, robustness and computational efficiency of our framework. We plan to apply this framework to magnetoencephalography (MEG) data together with MRI-derived SC in our upcoming work, where comparing against heuristic approaches under realistic experimental conditions will provide more meaningful and comprehensive insights, and demonstrate how our approach can provide complementary contributions to existing connectivity analysis techniques.

**Q2:** One crucial information to understand the manuscript is how the ground truth models are constructed and implemented. There is some scattered information, but there is not enough to get an insight on what the ground truth model looks like and how to replicate the model: How many cortical/subcortical areas are there? How was the regional heterogeneity implemented across areas? Was there any systematic relationship between regional heterogeneity and asymmetry? In Table 2, it is mentioned that wi and Ii took a range of values. How was this implemented? Sequentially? Randomly? How the levels of asymmetry in connectivity matrices looked like and how relative error varied across areas? Indeed, it is not very clear to me how ground truth models were implemented across different global coupling values.

**A2:** Thank you for this important question. We recognize the need for clearer explanation of our ground truth model implementation. We have substantially expanded the **Materials and Method** section and added clarifications throughout the **Results**.

The detailed description of empirical SC and regional heterogeneity (Materials and Method) are updated as proper reference to previous studies(Line 161-166) [36 and 43].

Yes, there is a systematic relationship between these two measures: regional heterogeneity reflects the hierarchical organization of cortical areas, while asymmetry captures the directional flow of information within this hierarchy. The systematic relationship between regional heterogeneity and asymmetry has been discussed in the Results (Line 138-144) and Discussion (Line 681-690).

The detailed regional values of w_i and I_i are updated and described in Table 1 (Line 168-170) and Figure 3A.

The level of asymmetry of SC is measured (Figure 1D, Line 252-255).

The ground truth calculation is mentioned in Main Text (Line 294-298) and explained in detail in Materials and Method.

Relative Errors of $w_{i}$ and $I_{i}$ across regions at three cases ($G=0.1,0.8$ and $1.3$) are added in the Figure S4.

We also explain how Jacobian is calculated and its functional meaning in nonlinear dynamics in (Line 179-185). Details are in **Materials and Method**.

**Q3:** The results regarding the sampling intervals are interesting and the issue is often ignored in the literature. However, the results should be carefully interpreted. It is reported that for sampling intervals larger than 0.3 seconds, the error of recovery drops rapidly. This is much higher than typical temporal resolution of BOLD signals, which the authors already discussed in the manuscript. As far as I understood this is a very serious limitation for the model, given that the authors propose the framework to estimate the parameters from BOLD signals. Furthermore, many studies recover parameters from BOLD signals using substantially longer sampling intervals, which requires explanation. Therefore, it is important to know that if this limitation is specific to reconstruction approach they introduced and if so, why?

**A3:** We apologize for any misleading impression in our manuscript. We want to emphasize that our framework was not specifically designed for BOLD fMRI applications. In this study, we only considered simulated neural activity and did not apply our method to BOLD signals. The sampling interval analysis represents a general theoretical contribution applicable to any temporally sampled neural data, helping researchers understand the fundamental trade-offs involved in connectivity estimation from down-sampled time series. For future applications, we plan to consider MEG data, which would be more directly compatible with our current framework given its higher temporal resolution and more direct relationship to neural activity.

**Clarification on sampling interval effects.** The sampling interval effect occurs when there is a temporal resolution mismatch between the real neural activity (which we assume is dynamics with high temporal resolution) and the observed neural activity (sampled from the real neural activity at given intervals). This creates two challenges: (1) exponential scaling transforms the original structural connectivity into sampling interval dependent EC, causing weaker connections to decay while preserving only the strongest interactions, and (2) the matrix logarithm transformation amplifies noise and can yield non-unique solutions to recover the original structural connectivity at longer intervals.

**Interpretation of recovery error.** The recovery error describes how well we can infer true asymmetric SCfrom interval-dependent EC. Performance drops rapidly beyond 0.3s because the mathematical transformation becomes unreliable due to noise accumulation and matrix logarithm limitations. Notably, previous studies have reported similar evolution patterns. Specifically, they found that compared to direct SC connections, the integration of SC in a diffusion manner showed higher correlation with pulse-response probability/amplitude measured from sEEG signals [66]. This suggests that neural signal transmission follows integrated pathways rather than direct anatomical connections, and that considering the cumulative effects of connectivity (similar to our mathematical framework) better captures the underlying neural dynamics. The integration approach accounts for how signals propagate through multiple pathways and accumulate over time, which aligns with our finding that sampling interval effects become critical when attempting to recover the true connectivity structure from observed dynamics.

**Regarding other studies using longer sampling intervals.** The key differences lie in our fundamental approach which differs from previous work in two important ways:

First, while previous studies focused on parameter estimation with fixed dMRI-based symmetric SC, they ignored the asymmetric components of true SC (which are not captured by dMRI) that might induce bias in the estimation. Our Figure 3 work is based on simultaneously separating effective heterogeneity and asymmetric SC components before reconstructing heterogeneous parameters. This decomposition allows us to better understand how the symmetric and asymmetric SC contribute to the observed dynamics.

Second, most existing EC methods can only estimate EC for a given time interval (typically based on BOLD signals after hemodynamic transformation), and thus the relationship of such estimated EC to the underlying SC remains unknown. In contrast, our framework explicitly models how temporal downsampling transforms the original SC into EC and attempts to recover the original SC underlying high-resolution dynamics through mathematical inversion (Eq. 18). We aim to decompose connectivity components that more closely approximate the true SC by "undoing" the effects of temporal downsampling. This inversion becomes ill-conditioned at longer intervals, creating performance limitations specific to methods that attempt to recover the original connectivity rather than simply estimate interval-specific EC. We have revised the manuscript to make these points clearer and avoid similar misunderstandings [Line 765-770, Line 592-595].

**Future Application to BOLD Signal.** While longer intervals in BOLD include both slow hemodynamics and downsampling effects, an explicit relationship between BOLD-estimated EC and original SC has not been established. We plan to address the complex relationship between sampling intervals and hemodynamic effects in BOLD data in future work. For example, by comparing MEG-based EC (which isolates downsampling effects on neural activity) with BOLD-based results, we may disentangle these confounding factors and establish clearer connections between BOLD-estimated EC and underlying SC.

**Q4:** The authors report that some parameters’ recovery in reconstruction is poor for both low and high values of global coupling. It is nicely pointed out that this is caused by nonlinear regions in firing rate change function. However, this implies another important limitation of the framework, which should be discussed in the manuscript. For example, it is known that the best fit between simulated and empirical values appear near the critical point, which is also caused by the nonlinear regions in firing rate functions. This means that the framework may perform well if the effects of complex, nonlinear interactions are minimal or absent, which undermines one of the main advantages of using dynamic mean field model. In such cases, one could use much simpler. phenomenological models such as multivariate Ornstein-Uhlenbeck model or an autoregressive model without losing much information.

**A4:** We thank the reviewer for this important concern. We have conducted additional analyses to address the relationship between nonlinearity and parameter estimation.

We characterized the bifurcation structure following a previous work, which reports that optimal empirical fit occurs before the second bifurcation point [40]. To test the effects of estimated parameters on nonlinear dynamics, we used our reconstructed parameters, and re-simulated neural activity, calculated model-simulated FC and evaluated FC correlations with the original FC in the ground truth (Figure S4).

Our results show that even in nonlinear regimes where parameter estimation is challenging, our framework successfully captures essential FC patterns (Figure S4B, solid line, averaged $corr=0.84$ at $G=1.3$ where the second bifurcation occurs). Re-simulation using estimated effective heterogeneity and asymmetric SC successfully reproduces the FC patterns (Figure S4B, dashed line). The reconstruction performance of external input $I_{i}$ is not robust but does not significantly influence the ability to capture FC patterns, and the variability in $I_{i}$ estimation is consistent with previous study that also reported inaccurate $I_{i}$ estimation [36].

Importantly, our reconstruction framework using linearized approximation of nonlinear models enables us to consistently capture the effective heterogeneity (influenced by both heterogeneous parameters and regional nonlinearity as $G$ varies) and the asymmetric SC (independent of nonlinearity). These mechanistic insights cannot be obtained from simpler phenomenological models, highlighting the unique value of our approach by decomposing EC into effective heterogeneity and asymmetric SC.

We have added discussion clarifying when our approach offers advantages over simpler alternatives (Line 740-752).

**Q5:** The authors also propose that they reconstruct the parameters given that the inhibitory populations are hidden. Similar to the previous point, this approach should be discussed with its limitations. For example, model C, that is adapted from Demirtas et al. [ref. 38], leads to oscillatory dynamics due to high feedforward and feedback inhibition between excitatory and inhibitory populations. In this case, nonlinearities in the model play a unique role that could not be replaced by simpler linear models. This would be unlikely captured without explicitly modeling inhibitory populations. Similarly, the circuit model B that the authors mentioned is adapted from Deco et al., 2021 [ref. 37] also uses a balanced architecture including inhibitory feedback to keep rates at spontaneous rate. Indeed, this paper has a focus on investigating the role of time constants (tau) and the role of excitability in relation to excitatory and inhibitory neurotransmitters. Mapping between these parameters both weakens the physical interpretation and the benefits of using a nonlinear model.

**A5:** We appreciate this important concern about the biological validity of our approach when applied to E-I models.

Our linearization approach is not meant to replace nonlinear models in all scenarios, but rather to provide an effective framework for connectivity and heterogeneity reconstruction when inhibitory populations are not directly observable. We fully acknowledge that feedback inhibition control (FIC) and the unique role of E-I populations (such as balanced amplification and oscillatory dynamics) are critical features that cannot be captured by linear approximations.

Previous studies on EC have found that the best fit to empirical data occurs at the edge of Hopf bifurcation, representing a regime where stable dynamics coexist with oscillatory behavior [65]. To address the concern, we have conducted additional analyses to test our reconstruction performance in Model C near bifurcations both with and without FIC (Figure S6). We characterized the bifurcation points where optimal fit between model FC and empirical FC occurs (without FIC) [65] and where the model becomes unstable at Hopf bifurcation (with FIC) [aligning with SI in reference 38]. We then tested whether our decomposed effective heterogeneity and asymmetric SC components can reproduce the FC patterns from the excitatory population when re-simulate the neural activity with the estimated parameters using Eq. S4 (Figure S6). Note that this analysis differs from Model A (Figure 3) because detailed parameter reconstruction cannot be performed in Model C due to unobserved inhibitory populations.

Our results demonstrate that even when the E-I model operates in highly nonlinear regimes near bifurcations and exhibits oscillatory behavior (both with and without FIC), our framework can still decompose EC into effective heterogeneity and asymmetric SC components and achieve good performance in reproducing FC patterns. While most EC studies face challenges in distinguishing E/I populations, our work provides a practical approach for understanding how E-I dynamics are manifested in EC. We have added discussion clarifying the scope and limitations of our approach when applied to realistic E-I models operating in nonlinear regimes (Line 746-752).

**Minor/Major Comment:**

**Q6:** There are a lot of repetitive/redundant sentences and themes throughout the manuscript, which makes it difficult to follow. I recommend the authors to revise the overall structure of the manuscript and to be more concise. I also note that there is an extensive reporting of methods in results section. It is often necessary a computational study, but in this case, I found it very difficult to distinguish between results and methods. It would be better to keep only the information that is essential to better make sense of the results and organize rest in materials and methods section.

**A6:** Thank you for this feedback. We have thoroughly revised the manuscript to address these issues: we eliminated redundant sentences and themes throughout the manuscript, streamlining each section to avoid unnecessary repetition. We restructured the Results section by moving detailed methodological descriptions to the Methods section. The Results now focus exclusively on findings and their interpretation, retaining only the essential context needed to understand the results. We also improved section transitions and added clearer subheadings to enhance readability and logical flow.

**Minor Comments:**

**Q7:** In line 375, Table 2 is referred for the definitions of Model A and Model B. However, this information is given in Table 1 and Table 2 seems to present fixed model parameters.
**A7:** The text previously at Line 375 now properly refers to Method section (Line 377). Table 1 provides the parameters (including heterogeneous parameters $w_{i}$ and $I_{i}$) needed for detailed model reconstruction, as described in Methods sections (Eqs. 28-30). We have updated all relevant descriptions to ensure proper cross-referencing between tables and text.

**Q8:** The authors used J to denote Jacobian matrix. However, there are other parameters in the model such as the constant JN (e.g. appearing in equations 3, 5, 7…) and later to denote local recurrent strength (JEE) . This makes it difficult to follow the equations and cause confusions.

**A8:** Thank you for pointing out this notation confusion. We have revised the notation system to improve clarity:

We have eliminated the overlapping use of "$J$" by incorporating the NMDA synapse coupling constant $J_{N} = 0.2609 (nA)$ directly into the regional recurrent strength parameter $w_{i}$. This change serves two purposes: (1) it removes the notation conflict so that $J$ only denote the Jacobian matrix, and (2) it provides biological meaning to $w_{i}$ as the recurrent NMDA synaptic excitation strength of area $i$. (Line 168 and Table 1).

In the section IV of the revised manuscript, $J_{S^{*}}$ is the Jacobian matrix of Model C considering both excitatory and inhibitory populations. Because each cortical region has two populations (E/I), the full Jacobian matrix $J_{S^{*}}$ has $2N$ dimensions with four $N$-dimension blocks: $\left[ \begin{matrix} J_{EE} & J_{EI} \\ J_{IE} & J_{II} \end{matrix} \right]_{2N\times2N}$. The top-left block $J_{EE}$ represents the long-range excitation-to-excitation connectivity across $N$ regions, while $J_{EI}$, $J_{IE}$ and $J_{II}$ are diagonal matrices since only excitatory populations participate in inter-regional communications. We have updated this section with more detailed explanations for clarity (Line 539-545).

We also have changed the covariance matrices between the populations, e.g. $C_{EE}$ in previous manuscript to $COV_{EE}$ to aviod any confusion to the asymmetric SC (Line 1052).

We believe these changes improve the mathematical clarity and readability of our work.

**Response to Reviewer #2:**

This manuscript presents a modified version of the previously published DDC method, extending it by incorporating node heterogeneity into the framework. The authors conduct a thorough theoretical and numerical analysis across three neural network models to examine both the strengths and limitations of the proposed approach. Several insightful observations are made throughout the study.
This is a compelling study that investigates the role of node heterogeneity in network dynamics. The derivation steps are clear, and the underlying assumptions are well-articulated and convincing. However, I believe the method’s potential has not yet been fully realized. In particular, it remains unclear how the mathematically defined heterogeneity translates to neuroscience-relevant phenomena. I elaborate on this point below.

**Response:** Thank you for your positive evaluation of our work and valuable suggestions for revision.

**Major Comments:**

**Q9:** The concept of effective node heterogeneity would benefit from being more directly linked to empirical neural recordings, rather than being treated as an abstract mathematical property. From Equation 7, heterogeneity appears to arise primarily from variation in node-wise steady states, which is some graph structure rather than the node's intrinic properties. Consider extending this formulation to account for biologically meaningful properties such as differences in intrinsic time scales across cortical regions (e.g., [Murray et al.]) or variations in noise level (e.g. not the exact studying showing neural variability but related one [Ito et al.]). Ideally, the method would be applied to a multi-region neural dataset, where one could test whether the proposed heterogeneity measure h correlates with region-specific features such as time scale or neural variability. While such an analysis may be beyond the scope of the current manuscript, I strongly encourage the authors to pursue this in future work.
References

- Murray, J., Bernacchia, A., Freedman, D. et al. A hierarchy of intrinsic timescales across primate cortex. Nat Neurosci 17, 1661–1663 (2014). https://doi.org/10.1038/nn.3862
- Ito, Takuya, et al. "Task-evoked activity quenches neural correlations and variability across cortical areas." PLoS computational biology 16.8 (2020): e1007983.

**A9:** We greatly appreciate the reviewer's insightful comment and the valuable references provided, which offer important biological support for our concept of effective heterogeneity.

We have provided the relationship of effective heterogeneity $h_{i}$ and heterogeneous parameter in our revised Figure 1C. We also added more explanation on how regional heterogeneous parameters $w_{i}$ and $I_{i}$ contribute to the regional steady state and therefore reflected in our effective heterogeneity in Eq. 6.

The effective heterogeneity we find across different heterogeneous modeling studies serve as the common formula that can characterize the contribution of heterogeneous parameters in a unified mathematical framework.

We strongly agree with the reviewer's suggestion to test whether our heterogeneity measure $h_{i}$ correlates with node properties such as intrinsic timescales and neural variability using empirical dataset. In this way, our formula establishes a relationship between biological features and dynamical properties. We are actively pursuing this direction in our ongoing work with multi-region MEG/sEEG datasets, and further testing on macaque spiking dataset will provide empirical validation of the biological significance of effective heterogeneity. We include a discussion of these future directions and the biological interpretations in the revised manuscript (Line 808-811).

Interestingly, our preliminary simulation work has revealed distinct contributions of network asymmetry and effective heterogeneity to brain dynamics: preserving effective heterogeneity while symmetrizing SC maintains FC patterns but alters timescale variability across brain regions; removing effective heterogeneity directly disrupts the timescale hierarchy across regions. These findings suggest that asymmetry and effective heterogeneity play different roles in shaping FC and intrinsic timescales.

**Q10:** Consider including benchmark comparisons to more clearly demonstrate the added value of incorporating node heterogeneity. For example, compare reconstruction errors between the proposed method and the original DDC (or other functional connectivity methods) that do not account for heterogeneity.

**A10:** This concern is directly addressed in our "**Overall Response to Baseline/Benchmark Comparisons**" section above. As detailed there, we now provide systematic benchmark comparisons including: (1) reconstruction performance on networks without heterogeneity/asymmetry, (2) comparisons with approaches that ignore these key features, and (3) robustness testing across another EC estimation method. These baseline/benchmark comparisons clearly demonstrate the added value of incorporating node heterogeneity in our framework. The new results are presented in the Main Text (Figure 2) and Supporting Information (Figure S2 and S3). The method details are presented in [Materials and Method].

**Q11:** The section on temporal scaling feels somewhat disconnected from the main narrative, as it appears to have limited relevance to the core topic of node heterogeneity. It may be more appropriate to move this discussion to a later section.

**A11:** The effects on temporal scaling is important in empirical applications. We have revised the temporal scaling section and moved it to the last section (Section VI) as suggested. This reorganization better aligns with the main narrative focused on node heterogeneity, while the temporal scaling discussion now serves as an important methodological consideration for applying our framework to empirical data with different sampling rates.

**Q12:** The estimation of the matrix logarithm introduces technical challenges. Existence and uniqueness of a valid solution are highly dependent on the structure of the connectivity matrix. It may be useful to discuss approximation strategies that ensure the matrix logarithm yields a well-defined estimator under a broader range of conditions.

**A12:** We acknowledge the technical challenges associated with matrix logarithm estimation. In our current implementation, we use standard matrix logarithm computation without specific regularization strategies, though we have tested Tikhonov regularization with similar results. We recognize that employing approximation methods or other regularization techniques could be key to reducing estimation errors and ensuring robust solutions across different connectivity matrix structures. Therefore, we plan to address this fundamental limitation by utilizing MEG data (with high temporal resolution) combined with dMRI-based SC to identify effective heterogeneity and asymmetry that are more directly related to neural activity. For MEG data analysis, we will explore alternative regularized matrix logarithm approaches to improve estimation stability. The well-defined estimator will allow us to explain BOLD-based EC through the temporal convolution of MEG-based EC with both the sampling effect and the hemodynamic response functions, providing a more neurophysiologically accurate framework [66]. We have added this technical discussion to the Discussion section, acknowledging both the matrix logarithm challenges and our future regularization strategies [Line 778-783].

**Q13:** In Figure 3B, J_T is estimated directly from DDC, and J_0 is subsequently computed considering sampling effects using Equation 18. However, it is surprising that J_0 performs worse. This appears counterintuitive and may depend on noise levels or properties of the underlying connectivity matrix. Could the authors clarify this, perhaps by showing performance under noiseless simulations?

**A13:** We appreciate this suggestion for clarification. The worse performance in estimating $J_{0}$ from $J_{T}$ is due to the technical issue that matrix logarithm can hardly recover weak connections that are weakened by temporal convolution (mentioned above at A12). Our simulation strategy involved generating data at $dt=0.01s$ and then downsampling it before applying DDC to obtain $J_{T}$. Under this framework, $J_{T}$ consistently matched our theoretical derivation across essentially all downsampling intervals $T$, validating our sampling effect theory (Eq. 18]). This confirms that the EC estimated from downsampled neural activity $J_{T}$ is indeed a convolution of the true EC $J_{o}$.

The key point is that $J_{T}$ represents the EC estimated from the observed (downsampled) data, while $J_{0}$ represents our attempt to recover the true underlying SC (at 0.01s resolution) from the downsampled observations. The counterintuitive result that $J_{0}$ performs worse reflects the fundamental challenge: as downsampling increases, more temporal precision is lost because rapid neural activity changes are eliminated by the downsampling process (also affected by the matrix logarithm, Eq. 18). Therefore, the degraded performance of $J_{0}$ demonstrates the inherent information loss when attempting to reconstruct high-temporal-resolution dynamics from low-temporal-resolution observations.

This finding highlights that while we can accurately characterize how sampling affects EC estimation ($J_{T}$), recovering the original connectivity ($J_{0}$) becomes increasingly difficult as the temporal resolution gap widens due to the technical difficulty of matrix logarithm computation (mentioned in A12). We have added this clarification to better explain the distinction between estimating connectivity from observed data versus attempting to recover true underlying connectivity (646-649).

**Q14:** Could the authors comment on the method’s ability to estimate self-connections? This is an important aspect of real neural systems that is often difficult to recover.
**A14:** We appreciate this important question about self-connections. In Figure 3A we can potentially estimate the underlying recurrent strength $w_{i}$ which represents self-dynamics activation. At the regional level, $w_{i}$ represents the comprehensive effect of local circuit connections within a cortical region, capturing the functional self-regulatory capacity that reflects the aggregate effect of anatomical self-connections.

Actually, we can rewrite Eq.3 as: $x_{i}=GC_{ii}S_{i}+G\Sigma_{j}C_{ij}S_{j}+I_{i},$ where we represent $w_{i}$ as $C_{ii}$ scaled by $G$. In this case, estimating $w_{i}$ is equivalent to estimating the self-connection of region $i$.Therefore, our approach provides estimation of regional self-connections that are neurophysiologically meaningful. This property is important for interpreting results in the context of real neural systems. We highlight this distinction between effective self-dynamics $J_{ii}$ and anatomical self-connections in the revised manuscript (Line 374-377, Line 878-884).

**Minor Comments:**
**Q15:** Table 1: For each model, please provide explicit expressions for the steady-state solutions, as these would help readers connect node heterogeneity to the graph structure.

**A15:** We thank the reviewer for this important suggestion. We have added explicit mathematical expressions for the steady-state solutions in Table 2 (we exchanged Table 1 and Table 2), which clearly demonstrate how node heterogeneity and graph structure interact. We have added more details to include these explicit expressions and their interpretations (Line 202-209).

**Q16:** Figure 1 and related figures showing estimated C_{ij}: Please consider placing the estimated C_{ij} matrices side-by-side with the corresponding ground-truth W_{ij} matrices. This would provide an intuitive visual sense of how node heterogeneity influences estimation accuracy.

**A16:** We thank the reviewer for this suggestion of illustration. We have updated Figure 1, where the difference between $W_{ij}$ and $C_{ij}$ are plotted at Figure 1D to highlight their systematic directional differences.

**Response to Reviewer #3:**

**Summary**

This manuscript introduces a novel framework to simultaneously reconstruct regional heterogeneity and asymmetric connections in brain networks using neural activity and symmetric structural connectivity data — both commonly available in experimental settings like fMRI and diffusion MRI.

The method builds upon and extends the Dynamical Differential Covariance (DDC) approach, which was previously developed for homogeneous networks. The authors demonstrate that regional heterogeneity and directionality can be separated mathematically and inferred via temporal reconstruction (estimating the Jacobian from neural dynamics, decoding dynamical directionality) followed by spatial reconstruction (decomposing the Jacobian using symmetric connectivity constraints, decoding effective heterogeneity and asymmetric structural connectivity). They validate the method using realistic macaque brain network models, including excitatory-inhibitory population dynamics.

The reviewer thinks the findings are suitable for publication in the PLoS Computational Biology, and sophisticated calculations are mostly carried out thoroughly and well described. The reviewer has only a few points for the improvement of the manuscript.

**Major Points**

**Q17:** Introduction: The Introduction is thoroughly written, providing enough motivation for the methods. Would you just provide why you chose to based your development on DDC method? In other words, what was the advantage the original DDC has over other EC methods?

**A17:** We chose DDC as our foundation because it provides a statistically unbiased estimator of EC based on least square error derivation, offering a simple and straightforward statistical framework for EC estimation. Additionally, as addressed in our "**Overall Response to Baseline/Benchmark Comparisons**" section above, we now include comparison with an alternative EC estimation method to demonstrate the robustness of our heterogeneity-aware framework. The relevant explanation has been added in the revised manuscript (Line 263-264, Line 359-365).

**Q18:** Results: The result section is rich with extensive models. I do have following suggestions.

**A18:** Thank you for your positive feedback on the comprehensiveness of our results section.

**Q19:** For a broader neuroscience audience, would you elaborate a bit more on the meaning of Jacobian structure and its implications? Also, citing one or two references (perhaps textbooks on dynamical systems or nonlinear dynamics) in the calculation of Jacobian (eq. 5 and on) would be helpful for the novices in the dynamical systems approach to the neuroscience.

**A19:** We have added more detailed explanations in both Main Text (Line 179-185) and Materials and Method (Section 2.A) regarding Jacobian calculation and its neuroscientific implications for a broader audience. The additions include: (1) a clear explanation of how we linearize the model through Taylor expansion, (2) detailed neuroscience interpretation of Jacobian elements and (3) references to dynamical systems literature [40 and 45 in Text] to support readers in understanding the approach.

**Q20:** I don’t have a clear idea, but I feel Figure 1 could be improved to include more insights on the temporal / spatial reconstruction scheme, especially the spatial reconstruction.

**A20:** We appreciate the reviewer's suggestion to enhance Figure 1 with more insights on the temporal and spatial reconstruction scheme. We have updated Figure 1 to better illustrate both the spatial distribution of regional heterogeneity and the temporal reconstruction process. We believe this can provide clearer visualization of how our method captures spatial and temporal features of brain network dynamics.

**Q21:** Would you describe at least briefly, how you arrived at the specific parameters on the table 2, or perhaps provide a very brief summary of the biological meanings for the values of parameters?

**A21:** We thank the reviewer for this important question about parameter selection. We have added detailed explanations regarding our parameter choices in Table 1 (We exchanged Table 1 and Table 2). These parameter choices of $w_{i}$ and $I_{i}$ reflect established neuroscientific knowledge about cortical organization and regional specialization. The specific fixed values (measured or fitted from empirical data) are properly referred to previous studies (Figure 3A, Line 168-170) and detailed in Materials and Method.

**Q22:** For the defined rho square in line 313 of page 19, would you at least provide a very brief summary of what the definition means for self-confinement of the manuscript, rather than just referring to reference 44?

**A22:** We have added more detailed explanation to ensure the logic is clear and self-contained, and we moved the sampling interval effect part into the last section (Section VI). We now explain that rho square represents the equivalent variance used to control sampling effects in discrete-time observations (**Materials and Method, Section 2.H**). This provides readers with a clear understanding of the sampling transformation without requiring them to consult the reference.

**Q23:** I am probably missing something, would you provide a bit more detail on how you got to eq. 18 from eq. 16?

**A23:** We apologize for the unclear derivation. We have clarified the mathematical steps from Eq. 16 to Eq. 18. Starting from the relationship $J_{T}=\frac{e^{TJ_{o}}-I}{T}$ (Eq. 16), we rearrange $e^{TJ_{o}} = TJ_{T}+I$. Taking the matrix logarithm of both sides: $TJ_{o} = ln\left( TJ_{T}+I \right)$. Dividing by $T$ gives: $J_{o}=\frac{ln\left( TJ_{T}+I \right)}{T}$, which is Eq. 18. This derivation allows us to estimate the original continuous-time Jacobian $J_{o}$ from the sampled discrete-time Jacobian $J_{T}$ by accounting for the sampling interval $T$. We have revised the text to make these mathematical steps explicit and clear for readers [Line 1132-1139].

**Q24:** In line 494 of page 29, I know reference 38 is referred to before, but would you cite it again as the source of the model C and eqs 23-28?

**A24:** Yes, we have added reference 38 before Model C is introduced and have revised the equation numbers accordingly (Line 534, Line 1008).

**Q25:** Discussion: I feel that the discussion part is thoughtfully written and has wide-ranging implications discussed well.

**A25:** Thank you for your positive feedback on the discussion section.

**Q26:** I just have one question. Would the findings and analysis schemes in this manuscript help in anyway to understand and analyze rapid BOLD dynamics, i.e., directionality changes between top-down pathway (information flow from higher order regions to lower order sensory area) and bottom-up pathway (Honey et al., 2017, Network Neuroscience 1, 339; or perhaps Bolt et al, 2022, Nature Neuroscience 25, 109)?

**A26:** Thank you for this insightful question. Technically, our results demonstrate that the estimated asymmetry can be independent of neural activity levels, while effective heterogeneity depends on activity levels and may reflect the directionality of neural information flow. Building on the work of Honey et al. (2017) and Bolt et al. (2022), our method could help quantify the dynamic switches between top-down and bottom-up pathways (or external and internal attention) by using effective heterogeneity as a measure of directional flow.

We plan several future explorations to address empirical dynamics and directionality changes:

First, we will consider using MEG data with dMRI-based SC to reconstruct EC, effective heterogeneity and asymmetric SC. This approach ensures high temporal resolution while avoiding the confounding effects of hemodynamics convolution in BOLD. In this empirical framework, we hypothesize that directional flow switching (or changes in directional flow proportions) may be primarily reflected in effective heterogeneity. The variance of regional effective heterogeneity—measured through windowed time series or by contrasting task vs. resting-state activity—could capture these flow transitions.

Second, we will validate our findings using sEEG data. There are datasets contain rich electrical stimulation response data, allowing us to investigate whether the effective heterogeneity we discovered and the sampling interval effects we identified have synergistic effects in explaining directional connectivity patterns.

Finally, we will compare MEG-based EC with hemodynamic convolution effects. Contrasting results from MEG and BOLD signals will help us understand the relationship between top-down/bottom-up flow switching, anatomical heterogeneity, asymmetry, and hemodynamic responses. This comprehensive approach should provide insights into how rapid directional changes in neural communication relate to the slower BOLD dynamics. In this framework, it is likely that rapid BOLD dynamics may be more sensitive to reflect directional information flow, as fast sampling can reduce the effects of sampling interval as inferred from our analysis in this work.

We acknowledge that our current focus on mathematical details and generalization may have overshadowed the broader neuroscientific implications of our work. We will emphasize applying our method to empirical data with greater attention to interpreting brain phenomena such as spontaneous top-down/bottom-up information flow, providing more interpretable and neuroscientifically meaningful results to our research community (Line 811-814).
