# Supplementary material for: Simultaneously determining regional heterogeneity and connection directionality from neural activity and symmetric connection
Source: PLoS Comput Biol. 2025 Oct 23;21(10):e1013612. doi: 10.1371/journal.pcbi.1013612 (PMC12574905; doi:10.1371/journal.pcbi.1013612)
Supplement: Attachment — Submitted filename: Response_to_Reviewers_auresp_2.docx [file pcbi.1013612.s012.docx]

Dear Editors,

Thank you for your editorial advice on our manuscript COMPBIOL-D-25-00885
“Simultaneously Determining Regional Heterogeneity and Connection Directionality from Neural Activity and Symmetric Connection” to PLOS Computational Biology.

We thank the reviewers for their constructive comments and suggestions. We have considered all of them carefully and addressed them in the revised manuscript. Below please find our point-by-point response to the reviewers.

Thank you for your attention and we look forward to your further editorial advice.

Sincerely yours,

Changsong Zhou (on behalf of the co-authors)

Email: [cszhou@hkbu.edu.hk](mailto:cszhou@hkbu.edu.hk)

Chair Professor, Department of Physics,

Director, Center for Nonlinear Studies

Director, Life Science Imaging Centre

Deputy Director, Institute of Computational and Theoretical Studies

Hong Kong Baptist University

Google Scholar:

<https://scholar.google.com.hk/citations?user=p3V8N-4AAAAJ&hl=en>

**Response to Reviewers**

**General Response**

We are pleased that the reviewers were largely satisfied with our previous revision. We are grateful to the reviewers for their further constructive feedback and valuable insights. In response to their feedback, we have further strengthened the overall narrative of the manuscript.

The specific revisions are outlined as follows:

**Response to Reviewer #1:**

 I thank the authors for their positive attitude, transparency and laborious efforts to address the concerns raised in the review. The manuscript is substantially improved after the revision. The inclusion of reconstruction performance under different assumptions supported the argument behind the main claim and emphasized its importance and relevance. Moreover, the ground truth model is much clearer in the revised version. They explained better how the method is connected to the previous models, and they addressed the issues regarding non-linear dynamics. I only have minor remarks that concern the narrative of the manuscript.

The relevance of sampling intervals is better explained, but I was surprised that it is much better explained in the rebuttal letter than in the manuscript. There are still some parts, for example the ones discussing BOLD deconvolution, that sound confusing.

The authors should revise the writing again. Despite not thoroughly scanning the manuscript on language issues and not being a native speaker, I came across various problems such as typos (e.g. line 293, “tolenrence”), grammar issues (e.g. line 346), difficult to read sentence structures (e.g. line 385) and use of a mixture of present and future (both for current and future work) tense throughout the manuscript.

Looking forward to seeing the extension of this work regarding the application of the method on MEG.

**Response:** Thank you for your positive evaluation of our work and valuable suggestions for revision. We have carefully revised our manuscript to address the following concerns raised by the reviewer:

**1. Language and Writing Quality Issues**

We have conducted a comprehensive checking and revision of the entire manuscript to address the language issues you identified. Specifically:

**Spelling errors.** We corrected all typos throughout the manuscript including:

“tolenrence” to “tolerance” (Line 293)

“parametrized” to “parameterized” (Line 373)

“aysmmetric” to “asymmetric” (Line 907)

“Reconstrution” to “Reconstruction” (Line 929)

“thershold” to “threshold” (Line 987)

“kernal” to “kernel” (Line 1109)

…

**Grammar issues.** We fixed grammatical problems such as subject-verb disagreement:

“The development in” to “Developments in” (Line 70)

“This increase can also be explained as” to “This increase can also be explained by the fact that” (Line 341-343)

“As compared” to “Compared to” (Line 1065-1067)

…

**Sentence structure.** We simplified complex and difficult-to-read sentences.

(Line 385, Line678, Line161)

**Tense consistency.** We systematically reviewed and corrected the mixture of present and future tenses throughout the manuscript:

“provides” to “have provided” (Line 70)

“which will be discussed” to “as discussed” (Line 197)

“The following sections will systematically demonstrate” to “The following sections systematically demonstrate” (Line 211)

“we will obtain an unbiased estimate” to “we can obtain an unbiased estimate” (Line 1046)

…

**2. Enhanced Explanation of Sampling Intervals**

Following your observation that the sampling interval relevance was better explained in our rebuttal letter than in the manuscript, we have incorporated and expanded these explanations directly into the revised manuscript (Line 766-774). The text now includes:

- Clearer biological significance of sampling intervals.
- More intuitive examples of how sampling rates affect connectivity estimation.
- Explicit discussion of implications for multi-modal data comparison.

**3. Clarity of BOLD Deconvolution Discussion**

We have substantially revised the sections discussing BOLD deconvolution to eliminate confusion (Line 794-810). The revised text now provides clearer explanations of:

- Artificial temporal dependencies introduced by hemodynamics response function.
- Two potential methodological solutions.

All revisions have been highlighted in the revised manuscript for your convenience. We believe these comprehensive changes have significantly improved the clarity and readability of our work while maintaining its scientific rigor and contribution.

**Response to Reviewer #2:**

I'm glad to see that the authors have addressed all my concerns and re-arranged the paper flow to be more readable.

**Response:** Thank you for your positive evaluation of our work.

**Response to Reviewer #3:**

I thank and appreciate the authors for making a thorough revision taking into accoount of the reviewer's commentss. I believe the manuscript has definitely become more informative and self-contained. I recommend the manuscript for the publication in PLOS Computational Biology.

**Response:** Thank you for your positive feedback on our research.
