# Supplementary material for: Simultaneously determining regional heterogeneity and connection directionality from neural activity and symmetric connection
Source: PLoS Comput Biol. 2025 Oct 23;21(10):e1013612. doi: 10.1371/journal.pcbi.1013612 (PMC12574905; doi:10.1371/journal.pcbi.1013612)
Supplement: Peer review history

# Peer review: Decision Letter 0

**DOI:** 10.1371/journal.pcbi.1013612.r001

**Authors:** Lyle J. Graham, Mikail Rubinov

16 Jun 2025

Simultaneously Determining Regional Heterogeneity and Connection Directionality from Neural Activity and Symmetric Connection

PLOS Computational Biology

Dear Dr. Zhou,

Thank you for submitting your manuscript to PLOS Computational Biology. After careful consideration, we feel that it has merit but does not fully meet PLOS Computational Biology's publication criteria as it currently stands. Therefore, we invite you to submit a revised version of the manuscript that addresses the points raised during the review process.

Please submit your revised manuscript within 60 days Aug 16 2025 11:59PM. If you will need more time than this to complete your revisions, please reply to this message or contact the journal office at ploscompbiol@plos.org. When you're ready to submit your revision, log on to https://www.editorialmanager.com/pcompbiol/ and select the 'Submissions Needing Revision' folder to locate your manuscript file.

Please include the following items when submitting your revised manuscript:

* A rebuttal letter that responds to each point raised by the editor and reviewer(s). You should upload this letter as a separate file labeled 'Response to Reviewers'. This file does not need to include responses to formatting updates and technical items listed in the 'Journal Requirements' section below.

* A marked-up copy of your manuscript that highlights changes made to the original version. You should upload this as a separate file labeled 'Revised Manuscript with Track Changes'.

* An unmarked version of your revised paper without tracked changes. You should upload this as a separate file labeled 'Manuscript'.

If you would like to make changes to your financial disclosure, competing interests statement, or data availability statement, please make these updates within the submission form at the time of resubmission. Guidelines for resubmitting your figure files are available below the reviewer comments at the end of this letter

We look forward to receiving your revised manuscript.

Kind regards,

Mikail Rubinov

Academic Editor

PLOS Computational Biology

Lyle Graham

Section Editor

PLOS Computational Biology

**Journal Requirements:**

1) We ask that a manuscript source file is provided at Revision. Please upload your manuscript file as a .doc, .docx, .rtf or .tex. If you are providing a .tex file, please upload it under the item type u2018LaTeX Source Fileu2019 and leave your .pdf version as the item type u2018Manuscriptu2019.

2) Please upload all main figures as separate Figure files in .tif or .eps format. For more information about how to convert and format your figure files please see our guidelines: 

https://journals.plos.org/ploscompbiol/s/figures

3) We notice that your supplementary Figures, and information are included in the manuscript file. Please remove them and upload them with the file type 'Supporting Information'. Please ensure that each Supporting Information file has a legend listed in the manuscript after the references list.

4) Please amend your detailed Financial Disclosure statement. This is published with the article. It must therefore be completed in full sentences and contain the exact wording you wish to be published.

1) State the initials, alongside each funding source, of each author to receive each grant. For example: "This work was supported by the National Institutes of Health (####### to AM; ###### to CJ) and the National Science Foundation (###### to AM)."

2) State what role the funders took in the study. If the funders had no role in your study, please state: "The funders had no role in study design, data collection and analysis, decision to publish, or preparation of the manuscript."

3) If any authors received a salary from any of your funders, please state which authors and which funders..

**Reviewers' comments:**

Reviewer's Responses to Questions

**Comments to the Authors:**

**Please note that one of the reviews is uploaded as an attachment.**

Reviewer #1: The manuscript by Chang and colleagues introduces a computation framework to estimate regional heterogeneity along with the directed connectivity from observed data. Specifically, they extend an existing framework that estimates the directed connections to include regional heterogeneity. Estimating the regional heterogeneity and directed (effective) connectivity is an important issue in large-scale computational modeling. Although in some studies the problem was highlighted, to my knowledge there are no studies directly attempting to address this issue. The overall methodological approach in the manuscript is sound. However, the authors need to resolve some major issues that can be categorized into 3 groups. First, the overall organization of the manuscript requires major revision, particularly to include all details to replicate the study. Second, although presented results show that the framework is robust, some critical questions to support the claims of the manuscript remains unanswered. Third, some of the claims in the discussion are not backed and limitations of the framework should be better emphasized in the manuscript.

Major Comment 1: Throughout the manuscript, relative error is used to quantify how well the framework performs. Although this quantity alone gives an idea about the relative performance of the fitting under different conditions, it lacks any proper reference. How the authors define the relative error good enough to support their claims? As far as I see, there are substantial differences in the scales of y-axis across different figures. The authors could use simplified models such as symmetrical and/or homogenous regional parameters as a baseline comparison. Optionally, it would be beneficial to compare the framework to more straight-forward ones such as heuristic approaches to estimate effective connectivity and heterogeneity in terms of recovery of ground truth parameters and computational cost.

Major Comment 2: One crucial information to understand the manuscript is how the ground truth models are constructed and implemented. There is some scattered information, but there is not enough to get an insight on what the ground truth model looks like and how to replicate the model: How many cortical/subcortical areas are there? How was the regional heterogeneity implemented across areas? Was there any systematic relationship between regional heterogeneity and asymmetry? In Table 2, it is mentioned that wi and Ii took a range of values. How was this implemented? Sequentially? Randomly? How the levels of asymmetry in connectivity matrices looked like and how relative error varied across areas? Indeed, it is not very clear to me how ground truth models were implemented across different global coupling values.

Major Comment 3: The results regarding the sampling intervals are interesting and the issue is often ignored in the literature. However, the results should be carefully interpreted. It is reported that for sampling intervals larger than 0.3 seconds, the error of recovery drops rapidly. This is much higher than typical temporal resolution of BOLD signals, which the authors already discussed in the manuscript. As far as I understood this is a very serious limitation for the model, given that the authors propose the framework to estimate the parameters from BOLD signals. Furthermore, many studies recover parameters from BOLD signals using substantially longer sampling intervals, which requires explanation. Therefore, it is important to know that if this limitation is specific to reconstruction approach they introduced and if so, why?

Major Comment 4: The authors report that some parameters’ recovery in reconstruction is poor for both low and high values of global coupling. It is nicely pointed out that this is caused by nonlinear regions in firing rate change function. However, this implies another important limitation of the framework, which should be discussed in the manuscript. For example, it is known that the best fit between simulated and empirical values appear near the critical point, which is also caused by the nonlinear regions in firing rate functions. This means that the framework may perform well if the effects of complex, nonlinear interactions are minimal or absent, which undermines one of the main advantages of using dynamic mean field model. In such cases, one could use much simpler. phenomenological models such as multivariate Ornstein-Uhlenbeck model or an autoregressive model without losing much information.

Major Comment 5: The authors also propose that they reconstruct the parameters given that the inhibitory populations are hidden. Similar to the previous point, this approach should be discussed with its limitations. For example, model C, that is adapted from Demirtas et al. [ref. 38], leads to oscillatory dynamics due to high feedforward and feedback inhibition between excitatory and inhibitory populations. In this case, nonlinearities in the model play a unique role that could not be replaced by simpler linear models. This would be unlikely captured without explicitly modeling inhibitory populations. Similarly, the circuit model B that the authors mentioned is adapted from Deco et al., 2021 [ref. 37] also uses a balanced architecture including inhibitory feedback to keep rates at spontaneous rate. Indeed, this paper has a focus on investigating the role of time constants (tau) and the role of excitability in relation to excitatory and inhibitory neurotransmitters. Mapping between these parameters both weakens the physical interpretation and the benefits of using a nonlinear model.

Minor/Major Comment: There are a lot of repetitive/redundant sentences and themes throughout the manuscript, which makes it difficult to follow. I recommend the authors to revise the overall structure of the manuscript and to be more concise. I also note that there is an extensive reporting of methods in results section. It is often necessary a computational study, but in this case, I found it very difficult to distinguish between results and methods. It would be better to keep only the information that is essential to better make sense of the results and organize rest in materials and methods section.

Minor Comment: In line 375, Table 2 is referred for the definitions of Model A and Model B. However, this information is given in Table 1 and Table 2 seems to present fixed model parameters.

Minor Comment: The authors used J to denote Jacobian matrix. However, there are other parameters in the model such as the constant JN (e.g. appearing in equations 3, 5, 7…) and later to denote local recurrent strength (JEE) . This makes it difficult to follow the equations and cause confusions.

Reviewer #2: Overall Evaluation:

This manuscript presents a modified version of the previously published DDC method, extending it by incorporating node heterogeneity into the framework. The authors conduct a thorough theoretical and numerical analysis across three neural network models to examine both the strengths and limitations of the proposed approach. Several insightful observations are made throughout the study.

This is a compelling study that investigates the role of node heterogeneity in network dynamics. The derivation steps are clear, and the underlying assumptions are well-articulated and convincing. However, I believe the method’s potential has not yet been fully realized. In particular, it remains unclear how the mathematically defined heterogeneity translates to neuroscience-relevant phenomena. I elaborate on this point below.

Major Comments:

- The concept of effective node heterogeneity would benefit from being more directly linked to empirical neural recordings, rather than being treated as an abstract mathematical property. From Equation 7, heterogeneity appears to arise primarily from variation in node-wise steady states, which is some graph structure rather than the node's intrinic properties. Consider extending this formulation to account for biologically meaningful properties such as differences in intrinsic time scales across cortical regions (e.g., [Murray et al.]) or variations in noise level (e.g. not the exact studying showing neural variability but related one [Ito et al.]) . Ideally, the method would be applied to a multi-region neural dataset, where one could test whether the proposed heterogeneity measure h correlates with region-specific features such as time scale or neural variability. While such an analysis may be beyond the scope of the current manuscript, I strongly encourage the authors to pursue this in future work.

- Consider including benchmark comparisons to more clearly demonstrate the added value of incorporating node heterogeneity. For example, compare reconstruction errors between the proposed method and the original DDC (or other functional connectivity methods) that do not account for heterogeneity.

- The section on temporal scaling feels somewhat disconnected from the main narrative, as it appears to have limited relevance to the core topic of node heterogeneity. It may be more appropriate to move this discussion to a later section.

- The estimation of the matrix logarithm introduces technical challenges. Existence and uniqueness of a valid solution are highly dependent on the structure of the connectivity matrix. It may be useful to discuss approximation strategies that ensure the matrix logarithm yields a well-defined estimator under a broader range of conditions.

- In Figure 3B, J_T is estimated directly from DDC, and J_0 is subsequently computed considering sampling effects using Equation 18. However, it is surprising that J_0 performs worse. This appears counterintuitive and may depend on noise levels or properties of the underlying connectivity matrix. Could the authors clarify this, perhaps by showing performance under noiseless simulations?

- Could the authors comment on the method’s ability to estimate self-connections? This is an important aspect of real neural systems that is often difficult to recover.

Minor Comments:

- Table 1: For each model, please provide explicit expressions for the steady-state solutions, as these would help readers connect node heterogeneity to the graph structure.

- Figure 1 and related figures showing estimated C_{ij}: Please consider placing the estimated C_{ij} matrices side-by-side with the corresponding ground-truth W_{ij} matrices. This would provide an intuitive visual sense of how node heterogeneity influences estimation accuracy.

References:

- Murray, J., Bernacchia, A., Freedman, D. et al. A hierarchy of intrinsic timescales across primate cortex. Nat Neurosci 17, 1661–1663 (2014). https://doi.org/10.1038/nn.3862

- Ito, Takuya, et al. "Task-evoked activity quenches neural correlations and variability across cortical areas." PLoS computational biology 16.8 (2020): e1007983.

Reviewer #3: The review is attached as a separate text file.

**********

**Have the authors made all data and (if applicable) computational code underlying the findings in their manuscript fully available?**

The PLOS Data policy requires authors to make all data and code underlying the findings described in their manuscript fully available without restriction, with rare exception (please refer to the Data Availability Statement in the manuscript PDF file). The data and code should be provided as part of the manuscript or its supporting information, or deposited to a public repository. For example, in addition to summary statistics, the data points behind means, medians and variance measures should be available. If there are restrictions on publicly sharing data or code —e.g. participant privacy or use of data from a third party—those must be specified.

Reviewer #1: Yes

Reviewer #2: Yes

Reviewer #3: Yes

**********

PLOS authors have the option to publish the peer review history of their article (what does this mean? ). If published, this will include your full peer review and any attached files.

If you choose “no”, your identity will remain anonymous but your review may still be made public.

**Do you want your identity to be public for this peer review?** For information about this choice, including consent withdrawal, please see our Privacy Policy .

Reviewer #1: No

Reviewer #2: **Yes: ** Yusi Chen

Reviewer #3: No

[NOTE: If reviewer comments were submitted as an attachment file, they will be attached to this email and accessible via the submission site. Please log into your account, locate the manuscript record, and check for the action link "View Attachments". If this link does not appear, there are no attachment files.]

**Figure resubmission:**

While revising your submission, please upload your figure files to the Preflight Analysis and Conversion Engine (PACE) digital diagnostic tool, https://pacev2.apexcovantage.com/. PACE helps ensure that figures meet PLOS requirements. To use PACE, you must first register as a user. Registration is free. Then, login and navigate to the UPLOAD tab, where you will find detailed instructions on how to use the tool. If you encounter any issues or have any questions when using PACE, please email PLOS at figures@plos.org. Please note that Supporting Information files do not need this step. If there are other versions of figure files still present in your submission file inventory at resubmission, please replace them with the PACE-processed versions.

**Reproducibility:**

?>

Attachment Submitted filename: PCOMPBIOL-D-25-00885 review.docx

# Author comment: Author response to Decision Letter 1

**DOI:** 10.1371/journal.pcbi.1013612.r002

19 Aug 2025

Attachment Submitted filename: Response to Reviewers.docx

# Peer review: Decision Letter 1

**DOI:** 10.1371/journal.pcbi.1013612.r003

**Authors:** Lyle J. Graham, Mikail Rubinov

18 Sep 2025

PCOMPBIOL-D-25-00885R1

Simultaneously Determining Regional Heterogeneity and Connection Directionality from Neural Activity and Symmetric Connection

PLOS Computational Biology

Dear Dr. Zhou,

Thank you for submitting your manuscript to PLOS Computational Biology. After careful consideration, we feel that it has merit but does not fully meet PLOS Computational Biology's publication criteria as it currently stands. Therefore, we invite you to submit a revised version of the manuscript that addresses the points raised during the review process.

Please submit your revised manuscript within 30 days Nov 18 2025 11:59PM. If you will need more time than this to complete your revisions, please reply to this message or contact the journal office at ploscompbiol@plos.org. When you're ready to submit your revision, log on to https://www.editorialmanager.com/pcompbiol/ and select the 'Submissions Needing Revision' folder to locate your manuscript file.

Please include the following items when submitting your revised manuscript:

* A rebuttal letter that responds to each point raised by the editor and reviewer(s). You should upload this letter as a separate file labeled 'Response to Reviewers'. This file does not need to include responses to formatting updates and technical items listed in the 'Journal Requirements' section below.

* A marked-up copy of your manuscript that highlights changes made to the original version. You should upload this as a separate file labeled 'Revised Manuscript with Track Changes'.

* An unmarked version of your revised paper without tracked changes. You should upload this as a separate file labeled 'Manuscript'.

If you would like to make changes to your financial disclosure, competing interests statement, or data availability statement, please make these updates within the submission form at the time of resubmission. Guidelines for resubmitting your figure files are available below the reviewer comments at the end of this letter.

We look forward to receiving your revised manuscript.

Kind regards,

Mikail Rubinov

Academic Editor

PLOS Computational Biology

Lyle Graham

Section Editor

PLOS Computational Biology

**********

Note: If the reviewer comments include a recommendation to cite specific previously published works, please review and evaluate these publications to determine whether they are relevant and should be cited. There is no requirement to cite these works unless the editor has indicated otherwise.

**********

**Reviewers' comments:**

Reviewer's Responses to Questions

Reviewer #1: I thank the authors for their positive attitude, transparency and laborious efforts to address the concerns raised in the review. The manuscript is substantially improved after the revision. The inclusion of reconstruction performance under different assumptions supported the argument behind the main claim and emphasized its importance and relevance. Moreover, the ground truth model is much clearer in the revised version. They explained better how the method is connected to the previous models, and they addressed the issues regarding non-linear dynamics. I only have minor remarks that concern the narrative of the manuscript.

The relevance of sampling intervals is better explained, but I was surprised that it is much better explained in the rebuttal letter than in the manuscript. There are still some parts, for example the ones discussing BOLD deconvolution, that sound confusing.

The authors should revise the writing again. Despite not thoroughly scanning the manuscript on language issues and not being a native speaker, I came across various problems such as typos (e.g. line 293, “tolenrence”), grammar issues (e.g. line 346), difficult to read sentence structures (e.g. line 385) and use of a mixture of present and future (both for current and future work) tense throughout the manuscript.

Looking forward to seeing the extension of this work regarding the application of the method on MEG.

Reviewer #2: I'm glad to see that the authors have addressed all my concerns and re-arranged the paper flow to be more readable.

Reviewer #3: I thank and appreciate the authors for making a thorough revision taking into accoount of the reviewer's commentss. I believe the manuscript has definitely become more informative and self-contained. I recommend the manuscript for the publication in PLOS Computational Biology.

**********

**Have the authors made all data and (if applicable) computational code underlying the findings in their manuscript fully available?**

The PLOS Data policy requires authors to make all data and code underlying the findings described in their manuscript fully available without restriction, with rare exception (please refer to the Data Availability Statement in the manuscript PDF file). The data and code should be provided as part of the manuscript or its supporting information, or deposited to a public repository. For example, in addition to summary statistics, the data points behind means, medians and variance measures should be available. If there are restrictions on publicly sharing data or code —e.g. participant privacy or use of data from a third party—those must be specified.

Reviewer #1: None

Reviewer #2: None

Reviewer #3: Yes

**********

PLOS authors have the option to publish the peer review history of their article (what does this mean? ). If published, this will include your full peer review and any attached files.

If you choose “no”, your identity will remain anonymous but your review may still be made public.

**Do you want your identity to be public for this peer review?** For information about this choice, including consent withdrawal, please see our Privacy Policy .

Reviewer #1: No

Reviewer #2: **Yes: ** Yusi Chen

Reviewer #3: **Yes: ** Joon-Young Moon

[NOTE: If reviewer comments were submitted as an attachment file, they will be attached to this email and accessible via the submission site. Please log into your account, locate the manuscript record, and check for the action link "View Attachments". If this link does not appear, there are no attachment files.]

**Figure resubmission:**

**Reproducibility:**

?>

# Author comment: Author response to Decision Letter 2

**DOI:** 10.1371/journal.pcbi.1013612.r004

6 Oct 2025

Attachment Submitted filename: Response_to_Reviewers_auresp_2.docx

# Editor report: Decision Letter 2

**DOI:** 10.1371/journal.pcbi.1013612.r005

**Authors:** Lyle J. Graham, Mikail Rubinov

12 Oct 2025

Dear Prof Zhou,

We are pleased to inform you that your manuscript 'Simultaneously Determining Regional Heterogeneity and Connection Directionality from Neural Activity and Symmetric Connection' has been provisionally accepted for publication in PLOS Computational Biology.

Before your manuscript can be formally accepted you will need to complete some formatting changes, which you will receive in a follow up email. A member of our team will be in touch with a set of requests.

Please note that your manuscript will not be scheduled for publication until you have made the required changes, so a swift response is appreciated.

IMPORTANT: The editorial review process is now complete. PLOS will only permit corrections to spelling, formatting or significant scientific errors from this point onwards. Requests for major changes, or any which affect the scientific understanding of your work, will cause delays to the publication date of your manuscript.

Should you, your institution's press office or the journal office choose to press release your paper, you will automatically be opted out of early publication. We ask that you notify us now if you or your institution is planning to press release the article. All press must be co-ordinated with PLOS.

Thank you again for supporting Open Access publishing; we are looking forward to publishing your work in PLOS Computational Biology. 

Best regards,

Mikail Rubinov

Academic Editor

PLOS Computational Biology

Lyle Graham

Section Editor

PLOS Computational Biology

***********************************************************

# Editor report: Acceptance letter

**DOI:** 10.1371/journal.pcbi.1013612.r006

**Authors:** Lyle J. Graham, Mikail Rubinov

PCOMPBIOL-D-25-00885R2

Simultaneously Determining Regional Heterogeneity and Connection Directionality from Neural Activity and Symmetric Connection

Dear Dr Zhou,

I am pleased to inform you that your manuscript has been formally accepted for publication in PLOS Computational Biology. Your manuscript is now with our production department and you will be notified of the publication date in due course.

The corresponding author will soon be receiving a typeset proof for review, to ensure errors have not been introduced during production. Please review the PDF proof of your manuscript carefully, as this is the last chance to correct any errors. Please note that major changes, or those which affect the scientific understanding of the work, will likely cause delays to the publication date of your manuscript.

Soon after your final files are uploaded, unless you have opted out, the early version of your manuscript will be published online. The date of the early version will be your article's publication date. The final article will be published to the same URL, and all versions of the paper will be accessible to readers.

For Research, Software, and Methods articles, you will receive an invoice from PLOS for your publication fee after your manuscript has reached the completed accept phase. If you receive an email requesting payment before acceptance or for any other service, this may be a phishing scheme. Learn how to identify phishing emails and protect your accounts at https://explore.plos.org/phishing.

Thank you again for supporting PLOS Computational Biology and open-access publishing. We are looking forward to publishing your work!

With kind regards,

Lilla Horvath

PLOS Computational Biology | Carlyle House, Carlyle Road, Cambridge CB4 3DN | United Kingdom ploscompbiol@plos.org | Phone +44 (0) 1223-442824 | ploscompbiol.org | @PLOSCompBiol
